# Astragalus polysaccharides: structure-immunomodulation relationships, multi-target pharmacological activities, and cutting-edge applications in immune modulation

**DOI:** 10.3389/fimmu.2025.1714898

**Published:** 2025-11-26

**Authors:** Bo Wang, Banglong Wu, Yingjuan Ma, Xiaofeng Liu, Lijun Tao, Limin Jia, Xiaoling Ding, Xuebing Zhou

**Affiliations:** 1 People’s Hospital of Ningxia Hui Autonomous Region, Ningxia Medical University, Yinchuan, China; 2 Department of Endocrinology, The Third Clinical Medical College of Ningxia Medical University, Yinchuan, China

**Keywords:** Astragalus polysaccharide, structure-activity relationship, immune regulation, cutting-edge applications, delivery innovations

## Abstract

Astragalus Polysaccharide (APS), the primary bioactive component of Astragalus, exhibits multi-faceted immunomodulatory properties. Its efficacy stems not from broad, non-specific stimulation but from the precise engagement of a network of cell surface immune receptors. This review synthesizes the critical structure-immunomodulatory network relationship of APS, positioning Toll-like receptor 4 as a central mediator. Key insights reveal that APS bioactivity is governed by a specific molecular weight window, critical monosaccharide ratios, and distinct glycosidic linkages. These structural features enable APS to interact with TLR4, potentially in collaboration with other pattern recognition receptors such as the Mannose Receptor and Dectin-1, to initiate integrated signaling. Future research must prioritize multi-omics and structural biology to map precise receptor-binding sites, establish robust standardization and quality control protocols, and advance translational clinical studies for APS-based adjuvant development. This work provides a strategic framework for advancing APS from a traditional remedy into a novel, mechanism-driven immunomodulatory agent.

## 1 Introduction

Plant-derived polysaccharides constitute a major class of biological response modifiers, renowned for their ability to interact with the immune system to either enhance host defenses or restore immunological homeostasis (1, 2). Among these, Astragalus polysaccharide (APS), a key bioactive component from *Astragalus membranaceus*, has garnered significant attention due to its demonstrated immunomodulatory, antitumor, anti-inflammatory, and metabolic-regulatory properties (3). A particularly important feature distinguishing APS from broad-spectrum immune stimulants is its ability to mediate effects through precise interactions with immune components, facilitating a finely-tuned regulatory response (4). However, this precision is complicated by a fundamental challenge: the substantial structural heterogeneity of APS, which includes variations in molecular weight, monosaccharide composition, glycosidic linkage patterns, and tertiary conformation. Therefore, a critical challenge in the field lies in deciphering how these specific structural features govern immune recognition and initiate downstream signaling, a knowledge gap that must be addressed for its rational development as a targeted immunotherapeutic agent.

The immunomodulatory capacity of APS is primarily attributed to its engagement with pattern recognition receptors (PRRs) (5). While Toll-like receptor 4 (TLR4) is the most extensively characterized receptor, serving a central role in activating the MyD88-dependent pathway, it is crucial to recognize that immune regulation inherently involves coordinated signaling across an integrated network (6–8). In line with this concept, mounting evidence suggests that other PRRs, including the mannose receptor (MR) and Dectin-1, may also function as targets for APS, an inference based on its monosaccharide profile and structural similarities to established ligands (9–11). Despite these indications, direct experimental validation of these interactions and a comprehensive delineation of the multi-receptor signaling landscape remain elusive, representing a considerable knowledge gap.

Although prior research has demonstrated the immunomodulatory effects of APS, highlighting their broad impact on immune organs, cells, and related diseases such as cancer and infections (12), a significant limitation of this existing body of work is that it has primarily emphasized phenomenological descriptions of immune responses. Specifically, it has not sufficiently delved into the structural basis of APS bioactivity, leaving a critical gap in understanding how specific molecular features (e.g., molecular weight, monosaccharide composition, glycosidic linkages) govern immune receptor engagement and signaling pathways. Furthermore, earlier reviews often lack integration of multi-target pharmacological mechanisms and cutting-edge applications, such as nanocarrier systems or vaccine adjuvants, which are crucial for translational progress. In contrast, this review is designed to address these limitations by systematically elucidating the structure-immunomodulation relationship of APS, positioning TLR4 as a central mediator and validating this interaction via modern analytical techniques. We further expand the scope by detailing multi-target activities beyond immunomodulation, including metabolic and anti-tumor effects, alongside innovative delivery strategies, thereby providing a strategic framework for advancing APS as a precision-based immunotherapeutic agent. This integrated approach not only clarifies the mechanistic foundation but also underscores the potential of APS in novel clinical applications, representing a significant advance in the field.

## 2 The influence of processing on the structure of Astragalus polysaccharides

Heterogeneity is the most distinctive feature of APS, arising principally from variations in the plant’s geographic origin, growth duration, post-harvest processing, and the specific extraction and purification protocols employed. The geographical origin of the Astragalus plant is a critical determinant of the resulting polysaccharide’s structural properties. Research comparing Astragalus from various producing areas (such as Inner Mongolia, Shanxi, and Gansu) reveals that although the fundamental monosaccharide profile and predominant glycosidic bond types of the water-soluble APS are similar, they exhibit marked variations in molecular weight (Mw), polydispersity, and the methylation level of uronic acid residues (13). These parameters are regarded as critical markers for differentiating APS based on their geographic provenance. Concurrently, these structural differences are thought to be linked to regional environmental conditions, including solar radiation, rainfall, and soil constituents. These factors modulate the enzymatic machinery responsible for polysaccharide biosynthesis in Astragalus, thereby inducing structural modifications in the final polysaccharide product (14). For example, Sheng et al. (13) conducted a systematic comparison of the structural profiles of Astragalus polysaccharides from various geographical sources, revealing a strong correlation between these structural disparities and biological activities, including antioxidant effects. Similarly, Zhang and colleagues (15) separated two homogeneous neutral polysaccharide fractions, designated APS-I (120 kDa) and APS-II (12 kDa). Both were identified as heteropolysaccharides with a high glucose content (exceeding 90%), but structural analysis revealed that APS-II adopted a highly branched architecture with a compact, spherical conformation, in contrast to the less branched, more extended linear chain morphology of APS-I. Capitalizing on these distinct features, the investigators evaluated their antitumor immunomodulatory activities, finding APS-II more potent at the same dosage.

Beyond geographic and cultivar variations, traditional Chinese medicine processing techniques significantly impact APS structure and activity. For instance, honey-frying causes profound changes, with studies confirming that honey-fried Astragalus polysaccharide (HAPS3a) undergoes significant structural alterations compared to raw APS (APS3a) (16). These alterations primarily include: (1) Reduced molecular weight due to partial hydrolysis of glycosidic bonds during processing (17); (2) Altered monosaccharide composition ratios from degradation or transformation (17, 18); and (3) Chemical modifications like Maillard reactions, introducing new functional groups and enhancing intermolecular hydrogen bonding, which affect solubility and conformation (17). These changes are considered the material basis for enhanced efficacy in functions like “tonifying Qi” and “moistening the lungs.” For example, honey-processed APS (HAPS) shows superior anti-inflammatory efficacy in murine colitis models (16). This phenomenon is not unique to APS; similar changes in monosaccharide composition and immune activity have been observed in other herbs like *Polygonum multiflorum* after processing (19). Collectively, these findings indicate that processing modulates bioactivity by precisely altering the primary structure of polysaccharides, providing a molecular perspective for understanding the mechanism of traditional processing. Other methods, such as drying, can also induce polymer aggregation or partial degradation, further underscoring that every step from field to final product shapes the structural and therapeutic properties of APS (20, 21).

**Figure 1:**
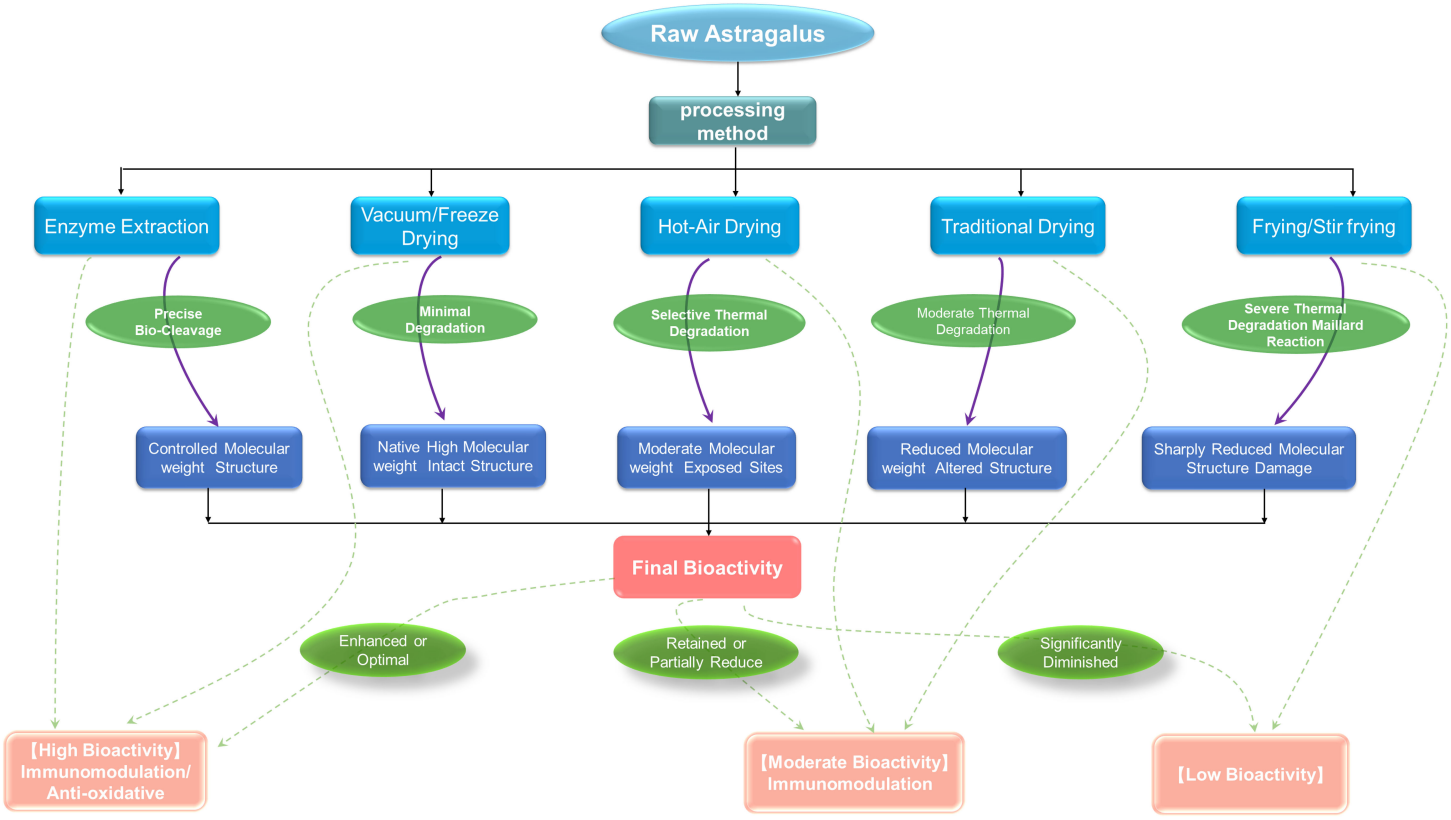
Processing methods and structure-activity relationship of Astragalus polysaccharides.

Furthermore, extraction and purification methods contribute to APS heterogeneity. Techniques such as hot water extraction, ultrasound-assisted, microwave-assisted, or enzyme-assisted extraction, along with purification steps like alcohol precipitation and chromatography, selectively enrich polysaccharides with varying molecular weights, charges, or solubilities, leading to distinct structural characteristics (3, 22, 23). This highlights the critical need to standardize and document the entire workflow for reliable structure-activity relationship (SAR) studies. The relationship between different processing/extraction methods of APS and the structure and activity are summarized in **Figure 1**.

## 3 Structure-immunomodulation relationships

**Figure 2:**
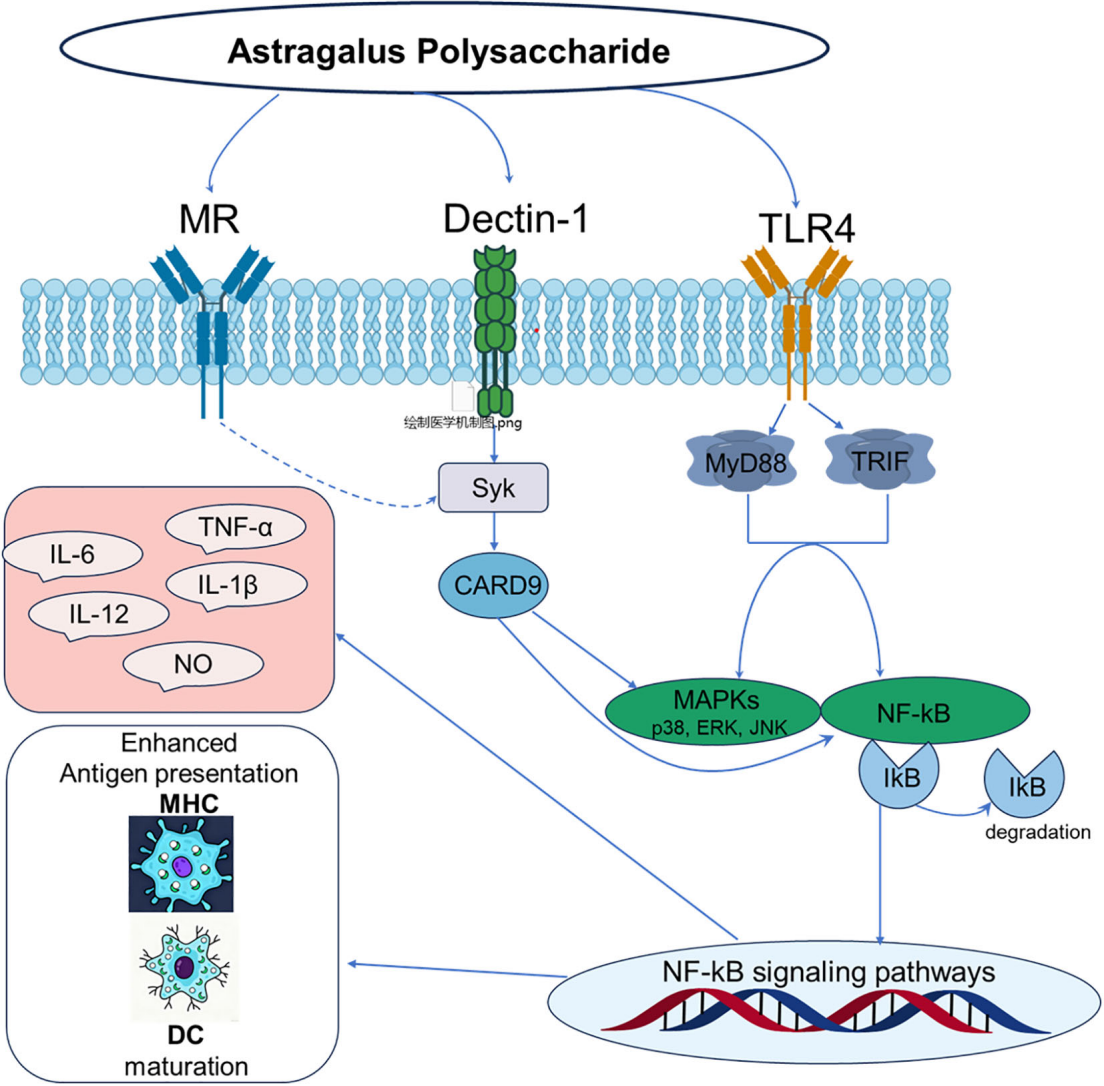
Molecular mechanism of immunomodulation by polysaccharides from Astragalus.

The innate immune system, which functions as the host’s primary defense against pathogens, is centered on pattern recognition receptors (PRRs) that detect pathogen-associated molecular patterns (PAMPs) (24–26). Among these PRRs, receptors such as Toll-like receptors (TLRs) and C-type lectin receptors (CLRs) act as immunological “sentinels” that perceive external danger signals (27). Substantial evidence indicates that APS, as a typical plant polysaccharide, is recognized by these sentinels; it initiates its immunomodulatory activity by binding to PRRs, thereby mimicking PAMPs to trigger downstream immune signaling cascades (28, 29). The molecular mechanism of APS immune regulation is shown in **Figure 2**.

### 3.1 Interaction of Astragalus polysaccharides with toll-like receptor 4

Among various PRRs, TLR4 is the most extensively studied and well-evidenced key receptor mediating the immunomodulatory effects of Astragalus polysaccharides. This receptor complex, comprising TLR4 and its accessory protein MD-2, is the principal sensor for Gram-negative bacterial lipopolysaccharide (LPS) and is pivotal in innate immune activation (30, 31). Accordingly, research confirms that APS functions as a TLR4 ligand, initiating downstream signaling cascades. Shao et al. (32) studies demonstrating that APS significantly upregulates TLR4 expression on immune cells such as macrophages and dendritic cells. A key piece of evidence is that anti-TLR4 monoclonal antibodies inhibit APS binding to macrophages, offering compelling support for a direct interaction.

Numerous studies consistently demonstrate that APS significantly upregulates TLR4 expression on the surface of immune cells such as macrophages and dendritic cells, activates the TLR4-mediated MyD88-dependent signaling pathway, leads to the recruitment of adaptor proteins MyD88 and TRAF-6, and activates transcription factors NF-κB and activator protein-1 (AP-1) (8, 33, 34). Following its binding to TLR4, APS initiates downstream signal transduction by recruiting the adaptor protein MyD88. MyD88 then sequentially activates interleukin-1 receptor-associated kinases (IRAKs) and tumor necrosis factor receptor-associated factor 6 (TRAF6), leading to the assembly of a signaling complex (26, 35). This signaling complex subsequently activates downstream mitogen-activated protein kinases (MAPKs), such as p38, ERK, and JNK, as well as the IκB kinase (IKK) complex. The activation of IKK catalyzes the phosphorylation and proteasomal degradation of the inhibitory protein IκBα, which liberates and activates the pivotal transcription factors NF-κB and AP-1 (36, 37). These activated transcription factors then translocate to the nucleus, driving the expression of pro-inflammatory cytokines and immunomodulatory molecules such as TNF-α, IL-6, IL-1β, and nitric oxide (NO) (38, 39).

It is important to emphasize that the majority of TLR4 involvement in APS activity is inferred from downstream signaling changes, antibody-blocking experiments, and receptor-expression modulation; direct biophysical confirmation of APS–TLR4/MD-2 binding (e.g., SPR, ITC) remains limited to date. Therefore, while the status of TLR4 as a core target of APS is well-established, it is crucial to note that current research largely relies on indirect measurements of downstream signaling to infer its role. Consequently, the critical questions of which specific structural features in APS mimic LPS to bind the TLR4/MD2 complex, and the associated binding affinity/kinetics, remain key subjects for deeper investigation.

### 3.2 Interaction between Astragalus polysaccharides and mannose receptor

The extracellular region of MR contains multiple tandem C-type lectin-like domains (CTLDs), with CTLD-4 and CTLD-5 being the key regions for binding carbohydrate ligands (40, 41). These domains act in concert to facilitate high-affinity binding to polysaccharide structures that possess multiple terminal mannose residues (42). It is well-established that numerous polysaccharides originating from fungi, yeast, and certain plant species exert their immunomodulatory effects via engagement with the MR. For instance, mannan from Candida albicans and specific polysaccharides from Ganoderma lucidum, both rich in mannose, have been identified as recognition ligands for the MR (6, 43, 44); this engagement can trigger cellular endocytosis, antigen presentation, and cytokine modulation (45). Given the presence of mannose in APS, it is a plausible candidate for MR interaction. Chemical analyses of APS reveal a heterogeneous monosaccharide composition that includes a significant proportion of mannose (9), providing a structural basis for this potential interaction. Supporting this, research has demonstrated that APS treatment upregulates the cell surface expression of the MR (10). This may be related to the direct or indirect activation of cells by APS, whereby MR expression is upregulated as a downstream effect of cell activation to enhance the cell’s ability to recognize other pathogens (46). However, the observed upregulation in expression is insufficient to establish MR as a direct receptor for APS; this could also be a downstream effect of general cell activation. This considerable knowledge gap delineates a clear research direction. Employing modern techniques to systematically validate the APS-MR interaction is therefore imperative, as it will not only refine our understanding of the immunomodulatory mechanism of APS but could also pioneer new pathways for developing highly targeted polysaccharide immunomodulators.

### 3.3 Interaction of Astragalus polysaccharides with dectin-1

Dectin-1, a member of the C-type lectin receptors (CLRs) family, is the principal receptor for fungal β-glucans, characterized by a β-(1,3)-linked backbone often with β-(1,6)-linked side chains (47–49). Recognition occurs via the extracellular C-type lectin-like domain (CTLD) in a calcium-independent manner, where the glucan chain interacts with a hydrophobic pocket (50–52). Ligand binding induces receptor clustering, phosphorylation of the intracellular ITAM-like motif, and recruitment of spleen tyrosine kinase (Syk) (53, 54). Activated Syk propagates the signal, leading to NF-κB activation via the CARD9-Bcl10-MALT1 complex, and can also elicit reactive oxygen species (ROS) production, NLRP3 inflammasome activation, and enhanced phagocytosis (55–57). A notable feature is the significant synergistic crosstalk that occurs between the Dectin-1 and Toll-like receptor (TLR, especially TLR2) signaling pathways. Simultaneous activation of both receptors leads to a more robust production of cytokines like TNF-α and IL-1β, facilitating a finely tuned immune response (29, 58).

Given the structural specificity of APS, Dectin-1 represents a promising potential target, particularly due to the presence of glucose configured into β-glucan-like structures. It is therefore postulated that APS may achieve synergistic amplification of its effects by simultaneously engaging both TLR4 and Dectin-1 receptors. However, while the involvement of TLR4 is well-documented, current research faces significant mechanistic gaps. Direct experimental evidence, such as quantitative binding affinity data for the APS-TLR4/MD-2 interaction or confirmation of binding to Dectin-1, is currently absent. This lack of direct evidence means the precise molecular patterns recognized by these receptors and the potential for synergistic signaling remain unclear. To address these gaps and construct a complete APS-PRR interaction map, future research should focus on a multi-faceted approach. First, obtaining structurally defined, homogeneous APS fractions through advanced separation techniques is essential. Subsequently, direct binding affinities (e.g., Kd values) and kinetics for receptors like TLR4/MD-2 and Dectin-1 must be quantified using biophysical methods like Surface Plasmon Resonance (SPR). These efforts should be combined with molecular docking simulations and site-directed mutagenesis to elucidate binding epitopes at an atomic level. Finally, systematic comparisons of well-characterized APS fractions in cellular models, using specific inhibitors or gene-knockout cells, are needed to delineate the individual and synergistic contributions of different PRR pathways to APS’s overall immunomodulatory effect. This comprehensive strategy is critical for advancing APS from a complex mixture to a precisely defined immunomodulatory agent.

### 3.4 Immune microenvironment reprogramming

**Figure 3:**
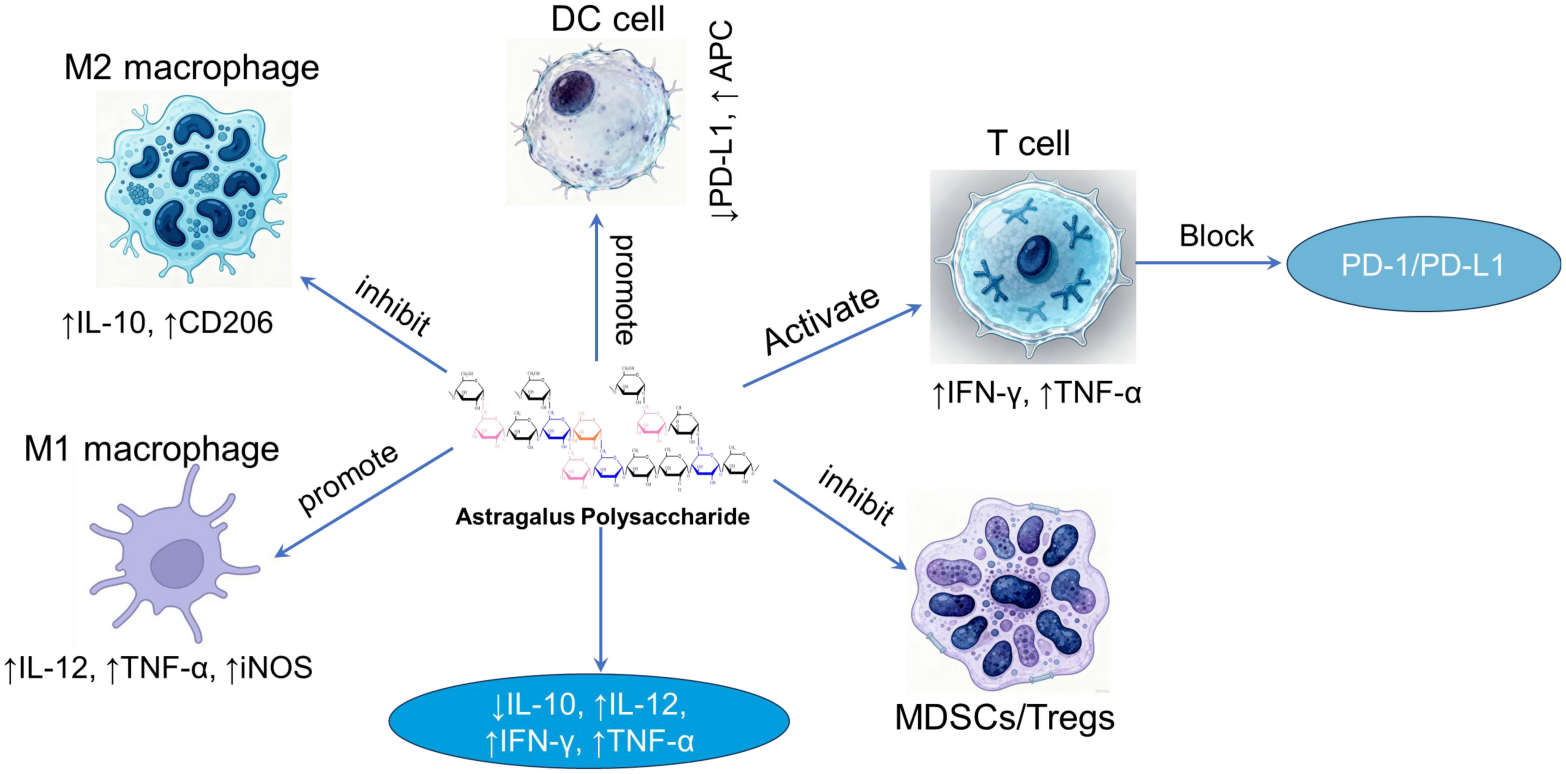
The relationship between macrophages, dendritic cells, T cells, and cytokines in the presence of APS.

Immune reprogramming” describes the process of altering immune cell identity and function through external intervention (59), providing a framework for natural product-based therapy. Research indicates that APS acts not as a simple immune booster but as a precise modulator that reprograms the immune microenvironment. APS directly or indirectly activates macrophages, dendritic cells, and other immune cells, binding to their surface receptors. This interaction promotes the proliferation and differentiation of immune cells, increases cytokine secretion, and alleviates immune suppression (**Figure 3**). These results provide a relevant evidence base for guiding the use of polysaccharides as adjuvants in cancer immunotherapy.

#### 3.4.1 Reversal of T cell exhaustion

In recent years, cancer immunotherapy, represented by Immune Checkpoint Inhibitors (ICIs) and Chimeric Antigen Receptor T-cell (CAR-T) therapy, has achieved revolutionary breakthroughs. However, a significant clinical challenge remains: a substantial subset of patients either fails to respond initially or acquire resistance. A pivotal mechanism for this limitation is T cell exhaustion (60, 61), which is intimately linked to the profoundly immunosuppressive nature of the tumor microenvironment (TME). APS can remodel the TME to counteract this exhaustion through multiple mechanisms. First, it modulates key immunosuppressive cell populations. Research demonstrates that APS can alter the differentiation and function of myeloid-derived suppressor cells (MDSCs) (62) and potentially attenuate regulatory T cell (Treg) recruitment, possibly via inhibition of Foxp3 expression (63). Diminishing the abundance and suppressive activity of these cells alleviates the burden on exhausted T cells, fostering a conducive milieu for functional recovery. Second, APS enhances antigen presentation. It potently promotes the maturation and functional capacity of dendritic cells (DCs), consequently boosting their antigen presentation ability (33, 64). Fully mature DCs deliver more robust T cell activation signals, a process that may promote the differentiation of T cells into effector phenotypes rather than exhausted states. Supporting this, studies in animal models indicate that co-administration of APS with chemotherapy drugs markedly enhances the infiltration of CD8^+^ T cells into tumor sites (65), shifting the balance between effector and suppressor cells. Enhanced recruitment of effector cells to the tumor site is a fundamental prerequisite for antitumor efficacy and likely shifts the balance between effector and suppressor cells within the TME, potentially overcoming the local immunosuppression.

A particularly compelling mechanism involves the direct targeting of the PD-1/PD-L1 pathway, a cornerstone of T cell exhaustion. An illuminating study revealed that APS can stimulate the endogenous generation of antibodies targeting PD-1. These antibodies inhibit the PD-1/PD-L1 interaction, leading to suppressed tumor progression (66). This suggests a novel paradigm where APS acts as a molecular mimic, eliciting a humoral response to indirectly facilitate checkpoint inhibition. Furthermore, other studies report that APS can directly reduce the expression levels of PD-L1 within the TME (33). Downregulation of this key ligand effectively diminishes the inhibitory signals received by T cells, thereby contributing to the relief from chronic exhaustion.

#### 3.4.2 Modulation of regulatory T cells

Regulatory T cells (Tregs), defined by the master transcription factor Foxp3, constitute a pivotal immunosuppressive subset essential for maintaining self-tolerance and controlling inflammation (67). However, their hyperactivation in settings like cancer suppresses productive immune responses, facilitating immune evasion (68, 69). The modulatory impact of APS on Tregs demonstrates a remarkable “context-dependency,” performing opposing immunoregulatory roles to restore immune homeostasis. In the context of cancer and immunosuppression, APS primarily acts to inhibit Treg function. For instance, APS has been reported to suppress the functionality of CD4^+^CD25high Treg cells in the microenvironment of human liver cancer (68). Similarly, research indicates that APS promotes tumor regression by inhibiting Treg activation (69, 70). This dampening of Treg-mediated suppression effectively “unleashes” cytotoxic T lymphocytes (CTLs), enhancing anti-tumor efficacy. Supporting this mechanism in a non-cancer context, APS was found to improve the host’s immunosuppressed state in a burn-induced sepsis model by inhibiting the negative immunomodulatory function of CD4^+^CD25high T cells, thereby mitigating septic progression (71). In these scenarios, the net result is the reversal of immunosuppression.

Conversely, under conditions of excessive immune activation and tissue injury, APS can promote Treg activity to curb inflammation. A study demonstrated that APS markedly ameliorated experimental colitis in rats by promoting the generation of Treg cells within the intestinal Peyer’s patches (72). Furthermore, APS may also reprogram Treg functionality; in a periodontitis model, it reduced the abundance of Foxp3^+^ Tregs while increasing the population of IL-10-producing Tregs, suggesting a sophisticated form of regulation that enhances anti-inflammatory capacity (73). Here, the fundamental outcome is the re-establishment of immune tolerance, positioning APS as an “immune stabilizer”.

### 3.5 Other potential receptors

NOD-like receptors (NLRs) are a class of cytosolic pattern recognition receptors (PRRs) characterized by a typical structure: an N-terminal effector domain, a central nucleotide-binding domain (NACHT), and a C-terminal leucine-rich repeat (LRR) domain responsible for ligand sensing (74). Among them, NLRP3 is a well-studied member activated by a wide array of stimuli, from pathogens to endogenous danger signals (75). A key mechanism underlying the immunomodulatory effects of APS appears to be the direct or indirect modulation of NLR signaling pathways, particularly the NLRP3 inflammasome, providing a molecular basis for its anti-inflammatory properties. This is evidenced by studies across different inflammatory disease models. In a mouse model of DSS-induced inflammatory bowel disease, APS significantly inhibited NLRP3 inflammasome activation, leading to reduced levels of mature IL-1β and IL-18 (76). Similarly, in an ovalbumin-induced allergic rhinitis model, APS alleviated nasal inflammation by suppressing NLRP3 inflammasome activation (77). The inhibitory action of APS is thought to operate at multiple levels of the NLRP3 activation cascade. Proposed mechanisms include the scavenging of reactive oxygen species (ROS)—a key trigger for NLRP3—through its antioxidant properties, the modulation of mitochondrial function, or direct interference with protein interactions within the inflammasome complex. However, the precise molecular targets and detailed mechanisms remain an important area for future investigation.

### 3.6 Structural features dictating immunological activity

**Figure 4:**
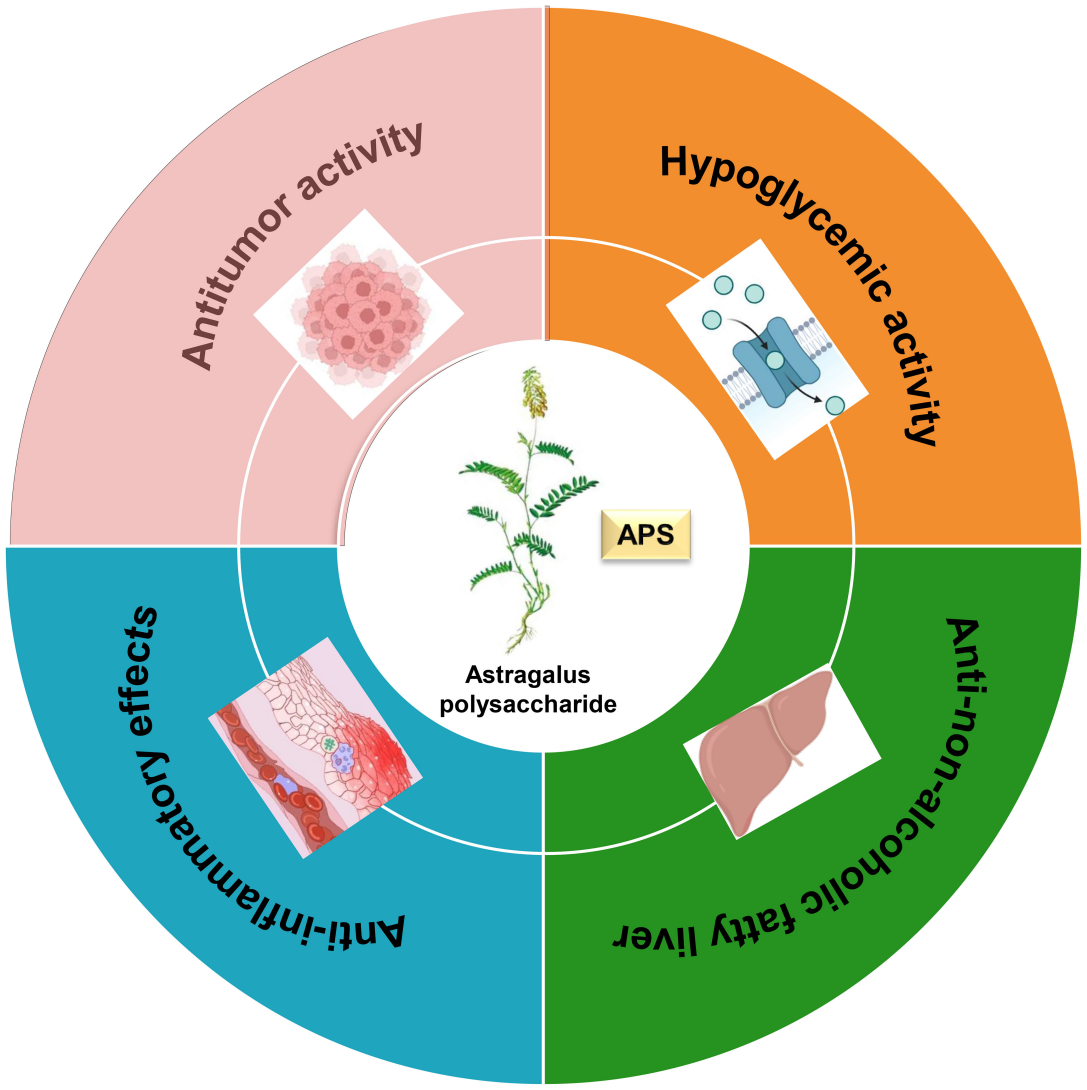
The biological activities of Astragalus polysaccharide.

The biological activity of APS is profoundly influenced by their specific chemical structural attributes, including molecular weight, monosaccharide composition, types of glycosidic linkages, branching architecture, and three-dimensional conformation (**Figure 4**). However, owing to the inherent structural complexity of APS, investigations explicitly correlating its structure with immunological activity remain limited. Nevertheless, synthesizing insights from the existing body of research is crucial for guiding future studies. Therefore, this section provides a discussion of the relationship between APS structure and its immunomodulatory effects, drawing upon available findings. The relationship between the structure of APS and immune regulatory activity is summarized in **Table 1**.

**Table 1:** Molecular weight, monosaccharide compositions and structural characteristics of Astragalus polysaccharides.

Fraction	Mw (kDa)	Monosaccharide composition (molar ratio)	Structural features	Mechanism	Biological activities	Ref
APS-I	>500	Glc: Gal: Ara: Rha: GalA=1.5:1:5.4:0.08:0.1	D-Glcp-(1→, →4)-D-Glcp-(1→, →2)-L-Rhap-(1→, D-Araf-(1→, →5)-D-Araf-(1→, →2,5)-D-Araf-(1→, →4)-D-Galp-(1 →	The phagocytic ability of macrophages, splenic lymphocyte proliferation, spleen T-lymphocyte proliferation, enhancing the toxic activity of NK cells (mouse, *in vitro*), spleen T-lymphocyte subsets CD3^+^ level significantly increased, and the CD4^+^/CD8^+^ level decreased, increased spleen and thymus indices	Immunomodulatory activity	(78)
APS-II	10	Glc:Gal:Ara:Rha:GalA=9:1:1.4:0.04:0.001	α-D-Glcp-(1→, →4)-α-D-Glcp-(1→, →6)-α-D-Glcp-(1→, →4,6)-α-D-Glcp-(1→, →3,4,6)-α-D-Glcp-(1→, → 2)-α-L-Rhap-(1→, α-D-Araf-(1→, →5)-α-D-Araf-(1→, →4)-β-D-Galp-(1 →	The phagocytic ability of macrophages, splenic lymphocyte proliferation, spleen T-lymphocyte proliferation, enhancing the toxic activity of NK cells (mouse, *in vitro*), spleen T-lymphocyte subsets CD3^+^ level significantly increased, and the CD4^+^/CD8^+^ level decreased, increased spleen and thymus indices	Immunomodulatory activity	(78)
AX-I-3b	7.87	Ara: Xyl: Glu=10.4:79.3:1.1	→2,3,4)-β-D-Xylp-(1→, →4)-β-D-Arap-(1→, →4)-β-D-Glcp-(1→	the organ index of spleen and thymus increased, Elevated levels of cytokines IL-2, IL-6, and TNF - α in mouse serum, The proportion of CD4^+^T cells and CD8^+^T cells in peripheral blood of mice increases, Increased expression of p53 in tumor cells upregulation of Bax/Bcl-2 ratio in tumor cells,	Antitumor and Immunological Activities	(79)
AMA-1-b-PS2	–	Ara: Fuc: Gal: Glu: Man: Rha: Xyl: GalA: GluA: GlcA=12.8:4.5:25.6:23.6:24.8:5.1: 0.7:1.5:1.5:1.4	β-D-(1→3)-galactans	Increased Peyer’s patch cells	Intestinal immune system-modulating	(80)
APSII	11.4	Xyl: Glu: Ara: Rha: Man: Gal=9.22:77.89:1:5.18:4.54:2.17	–	Increase the expression of surface markers MHCII, CD40, CD80, and CD86 on RAW264.7, induce NO production, and enhance cell phagocytic ability	Immunoregulatory effects	(81)
APS-IV	20.7	Glu	(1→4)-linked backbone with a (1→6)-linked branch every 10 residues	Stimulating the activity of mouse B cells without promoting T cell proliferation, Promote proliferation of spleen cells in BABL/c mice	Immunomodulatory activity	(82)
AMP	920.7	Glu: Ara: Gal=91.0:6.2:2.8	–	Stimulate RAW264.7 cells to produce NO and upregulate iNOS, TNF - α, IL-1 β, IL-6 mRNA expression through NF - κ B and MAPKs pathways	Immunomodulatory activity	(83)
RAP	1334	Rha: Ara: Glu: Gal: GalA=0.03:1.00:0.27:0.36:0.30	Backbone consists of →1,2,4)- Rhap, →1,4)-α- Glcp, →1,4)-α- GalAp6Me, →1,3,6)-β- Galp, with branched at O-4 of the →1,2,4)- Rhap and O-3 or O-4 of →1,3,6)-β- Galp	*In vitro* promotion of IL-1 β, TNF - α, IL-10, IL-12, and GM-CS production in human PBMCs	Immunomodulating effects	(84)
cAMPs-1 A	12.3	Fuc: Ara: Gal: Glu: Xyl=0.01:0.06:0.20:1.00:0.06	–	improve the macrophages pinocytosis and NK cells killing activity, Elevated proportion of CD4^+^and CD8^+^T cells in peripheral blood of tumor bearing mice,	Antitumor and immunomodulatory effects	(85)
ASP	2.1	Ara: Gal: Glu: Man=1.00:0.98:3.01:1.52	pyranose ring and α-type glycosidic linkages	Enhance the serum cytokine levels of H22 liver cancer cells, significantly inhibit their growth (TNF - α, IL-2, and IFN - γ) and immune cell activity (macrophages, lymphocytes, and NK cells)	Antitumor activity	(86)
APS-I	>2,000	Rha: GalA: Gal: Glu: Ara= 0.1:0.39:13.4:17.2:1	→3)-D-Glc-(1→, →4)-D-Glc-(1→, →6)-D-Gal-(1→, →3,4)-D-Gal (1→	Increase spleen and thymus indices	Immunomodulatory activity	(87)
APS-II	10.2	Rha: GalA: Gal: Glu: Ara= 0.14:0.14:9.6:24.04:1	D-Glcp-(1→, →4)-D-Glc-(1	Increase spleen and thymus indices, phenylalanine metabolism, cysteine and methionine metabolism, tricarboxylic acid cycle (TCA cycle) and arginine and proline metabolism	Immunomodulatory activity	(87)
APS-III	0.286	Rha: GalA: Gal: Glu: Ara= 0.375:0.375:18.8:90.5:1	→4)-D-Glc-(1→	Increase spleen and thymus indices	Immunomodulatory activity	(87)
APS-I	120	Glu: Gal: Man: Ara	–	TLR4-MyD88-NF-κB signaling pathway	Anti-tumor immunomodulatory effects	(15)
APS-II	12	Glu: Gal: Man: Ara	–	TLR4-MyD88-NF-κB signaling pathway	Anti-tumor immunomodulatory effects	(15)
APS1	106.5	Man: Rha: GalA: Glu: Gal: Ara= 0.79:1.70:3.28:7 9.07:6.63:8.56	→1,4)-β-Galp occasionally substituted by α-Araf at O-2 and/or O-3	Increase phagocytic activity and cytokine IL-6 and IL-1 β mRNA expression in RAW264.7 cells, MAPKs and NF-κB signaling pathways, promote the expression of IL-6, IL-1β and TNF-α	Immunomodulatory activities	(88)
APS2	114.5	Man: Rha: GalA: Glu: Gal: Ara= 1.61:6.05:10.21:6.34:27.39:48.39	long branches of α-Araf, which were attached to the O-3 of →1,6)-β-Galp of the backbone	Increase phagocytic activity and cytokine IL-6 and IL-1 β mRNA expression in RAW264.7 cells, MAPKs and NF-κB signaling pathways, promote the expression of IL-6, IL-1β and TNF-α	Immunomodulatory activities	(88)
APSN	4.97	Glu	a highly branched glucan with a →1,4)-α-D-glucopyranosyl main chain and side chains at the O-6 position	Inhibition of NF-κB pathway in RAW 264.7 macrophages promote endothelial cell proliferation and angiogenesis, and expression of VEGFA, EGF, and EGFL6	Immunomodulatory activities and anti-inflammatory activity	(89)

#### 3.6.1 Molecular weight

Molecular weight (Mw) is a fundamental property dictating polysaccharide bioactivity, primarily by modulating physicochemical characteristics such as solubility and viscosity, which in turn influence absorption and bioavailability. Evidence suggests that the most potent immunostimulatory polysaccharides often fall within a specific Mw window, potentially due to optimal solubility, tissue diffusibility, and capacity for productive receptor clustering. The relationship between Mw and activity has been systematically demonstrated for APS. Li and colleagues (90) fractionated crude APS into three distinct Mw ranges: a high-Mw fraction (>2000 kDa, APS-I), a medium-Mw fraction (~10 kDa, APS-II), and a low-Mw fraction (~300 Da, APS-III). Comparative analysis revealed a non-linear, parabolic relationship, identifying APS-II as the most immunomodulatory fraction. *In vivo* studies in an immunosuppressed mouse model showed APS-II was most effective at restoring immune organ indices and potentiating immune responses. It significantly outperformed other fractions in promoting splenic lymphocyte proliferation, inducing Th1-type cytokines (IL-2, IFN-γ), and enhancing innate immunity via macrophage phagocytosis and NK cell cytotoxicity (91, 92). The superior potency of the ~10 kDa fraction is attributed to its optimal physicochemical properties, such as good aqueous solubility and low viscosity, which promote systemic absorption and efficient engagement with immune cells (93, 94). In contrast, APS-I (>2000 kDa) showed weaker activity due to poor solubility and high viscosity, while APS-III (<1 kDa) exhibited minimal activity, suggesting a minimal chain length and specific 3D architecture are prerequisites for immune activation (87) (**Table 2**).

**Table 2:** Significant differences in the structure and activity of different Mw components of Astragalus polysaccharides.

Name	Mw	Monosaccharide composition	Immune activity intensity	Key evidence	Ref
APS-I	>2000 kDa	High Glc (Gal: Glc=17.2:1)	Medium	Promote macrophage phagocytosis, but weak cytokine induction	(87)
APS-II	10 kDa	High Gal (Gal: Glc =14.9:1), low GalA	optimal	NK cell activation, T/B cell proliferation, and IgG secretion	
APS-III	<1 kDa	extremely high Glc (Glc: Ar=90.5:1 a)	weak	Mainly focused on metabolic regulation, with limited immune activity	

This systematic comparison across orders-of-magnitude different fractions in APS research provides a clear functional differentiation that is less commonly encountered in studies of other polysaccharides like Lentinan. While Lentinan’s bioactivity is also known to relate to its molecular weight, extraction method, and conformation (95). The reported molecular weight range for Lentinan is broad, spanning from tens of kDa to several million Da; for example, studies have isolated fractions of 25.5 kDa, 306.2 kDa, and 605.4 kDa (96), or components as high as 6.8 x 10^6^ g/mol (97). The research focus for Lentinan differs, emphasizing the elucidation of its binding to specific receptors (e.g., Dectin-1) and subsequent signaling pathways (98, 99). Furthermore, the investigation of the low molecular weight APS fraction expands the understanding of small sugar bioactivity, an aspect often overlooked in traditional polysaccharide studies focused on high Mw polymers.

Therefore, while an optimal Mw range for APS activity is evident, its precise delineation remains tentative. The current suggestion of an optimal range (e.g., ~10 kDa) is inferred from limited comparisons, and a universal “optimal molecular weight window” for APS remains to be conclusively defined.

#### 3.6.2 Monosaccharide composition

A significant correlation exists between the immunomodulatory activity of APS and its specific monosaccharide composition. Although APS is a heterogeneous mixture, its bioactivity is critically determined by the quantitative ratios of its constituent monosaccharides, such as glucose, galactose, and arabinose. This principle is empirically supported by several studies. For instance, Guo et al. (9) demonstrated a positive correlation between the content of mannose, glucose, xylose, and fucose and the level of nitric oxide (NO) release, offering direct evidence that monosaccharide profile is a pivotal determinant of APS immunopotency. Further reinforcing this, Jiang and colleagues (100) verified that APS2 (a homopolysaccharide of arabinose) and APS3 (composed of Rha, Glu, Gal, and Ara in a distinct molar ratio) exhibited differing immunomodulatory potencies despite both stimulating splenocyte proliferation. The distinct activities of APS2 and APS3, which possess different monosaccharide compositions, provide compelling evidence that this parameter is a key factor underlying the variation in APS bioactivity. This principle of composition-dependent activity underscores why the elucidation of structure-activity relationships (SAR) is a central challenge in polysaccharide research. The highly complex and variable monosaccharide composition of well-known immunomodulatory polysaccharides like Ganoderma lucidum polysaccharides (GLPs) presents a significant obstacle to defining precise SAR (101). In this context, the structural profile of APS—characterized by a more homogeneous composition dominated by glucose and arabinose—offers a comparatively advantageous model for deciphering the link between specific structural motifs and their corresponding biological activities.

#### 3.6.3 Glycoside bond configuration

The bioactivity of a polysaccharide is critically determined by its structural features, most notably the types of glycosidic linkages and its degree of branching (102). For APS, a key structural motif has been identified: a glucan backbone consisting of α-(1→4)-linked D-glucose residues, which is ubiquitous in its bioactive fractions (103). Beyond the backbone, the presence and specific attachment points of branch chains are of paramount importance. Beyond the backbone, the presence and specific attachment points of branch chains are of paramount importance for immunological activity. A common structural pattern associated with high immunopotency is the presence of branch chains linked at the C6 position of the α-(1→4)-glucan backbone (104). The introduction of this branching enhances structural complexity, which is thought to modulate bioactivity by altering solubility, three-dimensional conformation, and the ability to engage in multipoint attachments with cellular receptors (105–107). Functional studies have correlated immunoactivity with the presence of specific glycosidic linkages, including, but are not limited to, →2,3)-α-L-Rha-(1→, →5)-α-L-Ara-(1→, →3,4)-β-D-Gal-(1→, →6)-β-D-Gal-(1→, →4)-α-D-Glu-(1→, and →3,4,6)-β-D-Glu-(1→ (90).

The structural logic of APS becomes clearer when contrasted with well-defined polysaccharides like Lentinan. Lentinan’s activity is attributed to a highly ordered structure—a β-(1→3)-glucan backbone with β-(1→6) branches—that forms a stable triple-helical conformation crucial for high-affinity binding to receptors like Dectin-1 (29, 108, 109). In contrast, the structure of APS exhibits significant “heterogeneity” and “complexity,” being a mixture of various linkage types encompassing both α- and β-configurations (86). This very complexity, however, constitutes its unique advantage. Unlike the specific, targeted action of β-glucans, the diverse structural motifs in APS can interact with a broader repertoire of immune receptors (e.g., TLR2, TLR4, MR), thereby enabling a “multi-pronged” mode of action that may result in a more comprehensive and balanced immunomodulatory outcome. Furthermore, this naturally complex mixture may better mimic physiological immune signals, regulating homeostasis in a more nuanced manner (110).

Notwithstanding this progress, a profound understanding of APS structure-activity relationships (SAR) remains challenging. The acquisition of homogeneous polysaccharide samples with defined structures, followed by comprehensive sequential and 3D structural analysis, continues to present a substantial obstacle that directly impedes a deeper mechanistic understanding.

## 4 Multi-target pharmacological activities beyond immunomodulation

**Figure 5:**
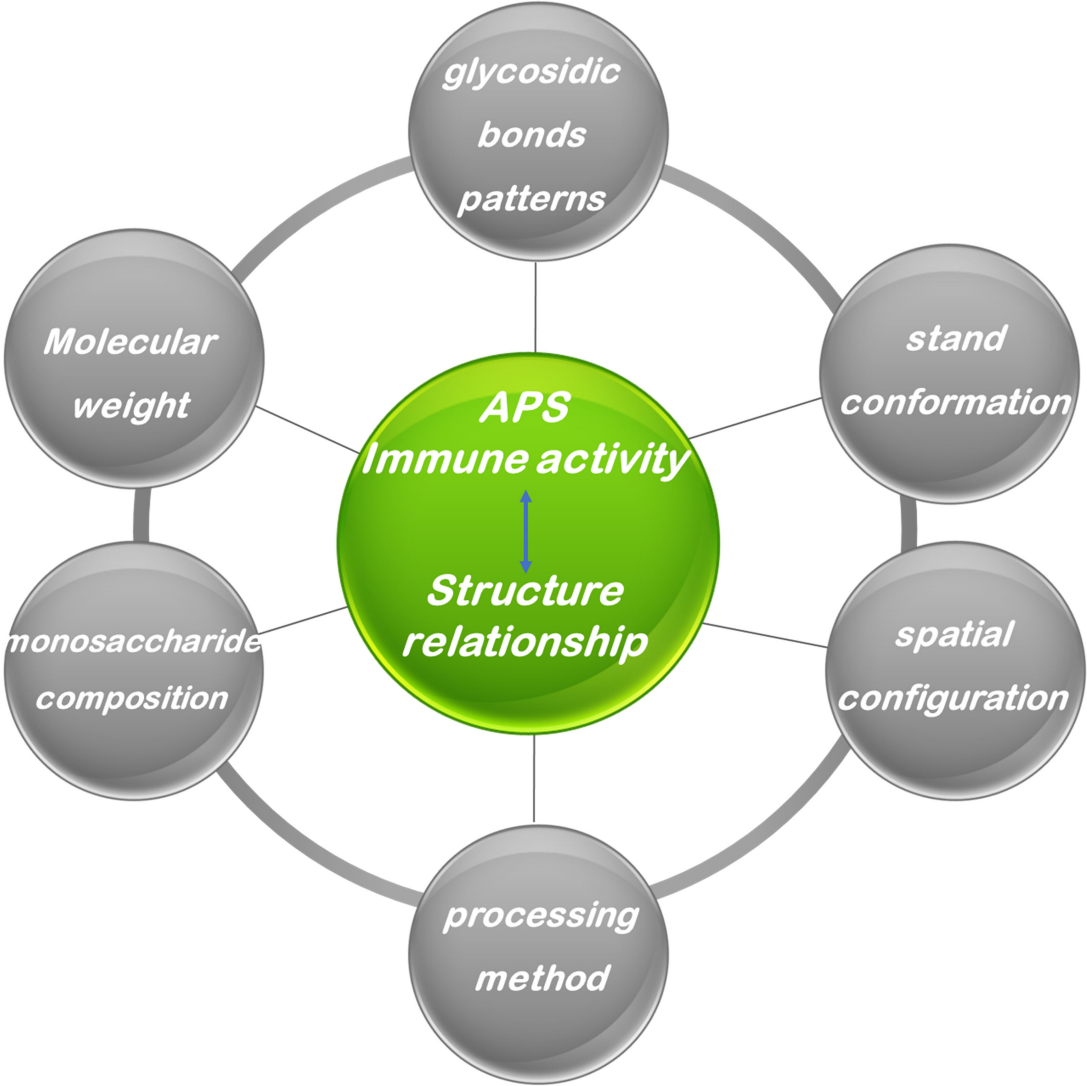
Relations between structure and immune effect of Astragalus polysaccharides.

Plant polysaccharides are natural macromolecules that have attracted widespread research interest due to their low toxicity and significant therapeutic potential. Among them, Astragalus polysaccharide is particularly notable for its diverse biological activities, which include hypoglycemic, anti-non-alcoholic fatty liver disease, anti-inflammatory, and anti-tumor properties (**Figure 5**). This multi-target therapeutic profile underscores the significant potential of APS for further pharmaceutical development and medical application.

### 4.1 Improvement of insulin resistance

Insulin resistance represents the central pathophysiological defect in T2DM. It has been demonstrated that APS effectively enhances insulin sensitivity and modulates glycemic homeostasis via several mechanisms independent of classical immune pathways. These mechanisms can be broadly categorized into direct insulin-sensitizing actions and broader metabolic improvements. A primary direct action is the potent activation of AMPK, a master regulator of cellular energy status. AMPK activation enhances glucose uptake and suppresses hepatic gluconeogenesis, consequently alleviating glucotoxicity (111, 112). Concurrently, APS augments insulin signaling by activating the Akt/GLUT4 axis to facilitate glucose disposal in peripheral tissues and by suppressing protein tyrosine phosphatase 1B (PTP1B), a key negative regulator of insulin receptor signaling (39, 113–115). Beyond direct signaling, APS elicits comprehensive metabolic improvements, particularly in the liver. It modulates the hepatic SIRT1-PGC-1α/PPARα-FGF21 signaling cascade to enhance fatty acid β-oxidation and ameliorate insulin resistance (116, 117). Separate research also indicates a role for the STAT5/IGF-1 pathway in improving hepatic insulin resistance (118). Furthermore, APS addresses the low-grade inflammation associated with metabolic dysregulation. It attenuates the secretion of pro-inflammatory cytokines such as IL-6, an effect mediated through direct action on metabolic cells, which indirectly contributes to improved insulin sensitivity (119).

### 4.2 Effects against non-alcoholic fatty liver disease

Non-alcoholic fatty liver disease (NAFLD) is a chronic hepatic disorder intimately linked to metabolic syndrome, with a spectrum ranging from simple steatosis to non-alcoholic steatohepatitis (NASH), which can progress to fibrosis, cirrhosis, and hepatocellular carcinoma. Driven by global pandemics of obesity and type 2 diabetes, NAFLD affects roughly one-quarter of adults worldwide (120). Critically, there are no FDA-approved pharmacotherapies for NAFLD, making the development of safe, effective treatments a pressing unmet need (121). Consequently, the development of safe and effective treatments for NAFLD represents a pressing unmet need in medical research.

The mechanisms of APS against NAFLD highlight its pleiotropic, multi-target effects beyond immunomodulation. APS directly targets key pathological processes in hepatocytes, including lipid accumulation, oxidative stress, and fibrosis progression. Furthermore, APS alleviates hepatic steatosis through several interconnected pathways. It facilitates the decomposition of intracellular lipid droplets by regulating the SIRT1-FoxO1-mediated autophagy axis (122). Additionally, APS mitigates hepatic glucolipid metabolic disturbances induced by stress through activation of the SIRT1/AMPK/PPARα/PPARγ signaling cascade. Concurrently, APS modulates crucial signaling pathways responsible for lipogenesis, including mTOR/4EBP1/S6K1, thereby suppressing hepatic *de novo* lipogenesis. Endoplasmic reticulum stress is a major contributor to hepatocellular injury during NAFLD progression. Additionally, APS protects against hepatocellular injury by attenuating endoplasmic reticulum stress, as evidenced by the inhibition of the GRP78/IRE-1/JNK pathway, which shields hepatocytes from apoptotic death and decelerates NAFLD advancement (117).

### 4.3 Direct antitumor effects

While considerable focus has been placed on the immunoadjuvant role of APS in oncology (11, 123), extensive *in vitro* evidence confirms that APS also exerts direct anti-neoplastic effects on cancer cells, manifesting as the inhibition of proliferation, induction of apoptosis, and suppression of metastatic potential (124, 125). These direct effects are mediated through multiple mechanisms. A primary effect is the induction of apoptosis and cell cycle arrest. APS promotes tumor cell apoptosis by modulating diverse signaling pathways, such as suppressing the Wnt/β-catenin pathway or altering the expression balance of p53 and Bcl-2/Bax proteins to trigger the caspase cascade (70, 126). Concurrently, APS suppresses aberrant proliferation by targeting cell cycle regulators like CCNB1 and CDC6, inducing arrest at the G0/G1 or G2/M checkpoints (127). APS potently inhibits the proliferation of carcinoma cells derived from the breast, lung, and liver (125, 128, 129). The underlying mechanisms involve the suppression of pivotal oncogenic signaling pathways, including EGFR, Notch, and JAK/STAT3 that drive proliferation and metastasis (130, 131).

Furthermore, APS effectively attenuates cancer metastasis by impeding the epithelial-mesenchymal transition (EMT) program and reducing the activity of matrix metalloproteinases (MMPs), thereby limiting invasive potential (126). A significant translational application of APS is its ability to potentiate chemosensitivity. Acting as a chemopotentiating agent, APS demonstrates marked synergistic anti-tumor activity when co-administered with conventional chemotherapeutics such as cisplatin and doxorubicin (65, 132). Proposed mechanisms encompass the modulation of drug efflux pumps (e.g., P-glycoprotein) to elevate intracellular drug levels (133); the regulation of autophagic flux to overcome chemoresistance (134), and the alteration of specific microRNA expression profiles (e.g., miR-195-5p) to re-sensitize cells to therapy (135).

### 4.4 Anti-inflammatory effects

Inflammation represents a fundamental defensive response of the innate immune system to insult or injury and is intimately linked to the pathogenesis of a wide spectrum of diseases. However, uncontrolled or chronic inflammation can be profoundly detrimental to health, contributing to the development of conditions such as inflammatory bowel disease, arthritis, neurodegenerative disorders, and cardiovascular disease. APS modulates inflammatory signaling via multi-target mechanisms, positioning it as a significant candidate for the development of natural anti-inflammatory therapeutics. In both LPS-stimulated macrophage cultures and *in vivo* animal models, APS markedly suppresses the release of key pro-inflammatory cytokines, including TNF-α, IL-1β, and IL-6, resulting in attenuated tissue inflammation and injury (136). These observations provide direct experimental support for the potential of APS as an effective anti-inflammatory agent. NF-κB is often termed the “master regulator” of inflammation, and its activation is a pivotal event initiating the transcription of a vast array of pro-inflammatory genes. Several studies (137, 138) have demonstrated that APS inhibits NF-κB activation by impeding the phosphorylation and proteasomal degradation of its inhibitory protein, IκBα. This results in the cytoplasmic retention of NF-κB, preventing its nuclear translocation and the subsequent initiation of pro-inflammatory gene transcription. Studies also report that APS can inhibit the phosphorylation of p38, ERK, and JNK, thereby blocking the transmission of upstream signals to nuclear transcription factors and cooperatively suppressing the inflammatory response. Furthermore, APS exerts its anti-inflammatory effects by inhibiting the JAK-STAT and PI3K/Akt signaling pathways. Oxidative stress can lead to an increase in intracellular oxygen free radicals and other reactive oxygen species, which can activate inflammatory responses, causing the infiltration of inflammatory cells and the release of inflammatory mediators. Research has found (139) that APS can activate nuclear factor erythroid 2-related factor 2 (Nrf2), promote its nuclear translocation, and induce the expression of downstream antioxidant proteins such as heme oxygenase-1 (HO-1), thereby scavenging reactive oxygen species (ROS), alleviating oxidative stress, and indirectly achieving anti-inflammatory effects through antioxidant actions.

Furthermore, studies have found that APS exhibits crosstalk with energy metabolism pathways. For example, the anti-inflammatory effects induced by APS in palmitate-treated RAW264.7 cells are dependent on the activity of AMP-activated protein kinase (AMPK), revealing that APS may influence the inflammatory process by modulating cellular energy metabolic status (10). The gut microbiota is regarded as the body’s “second genome,” and its homeostasis is crucial for host immune function. The anti-inflammatory effects of orally administered APS are largely achieved by remodeling the structure of the gut microbiota (140). Functioning as a prebiotic, APS can promote the growth of Lactobacillus and Bifidobacterium, inhibit harmful bacteria, and modulate microbial metabolites (e.g., short-chain fatty acids), thereby restoring intestinal barrier function, modulating systemic immune responses, and ultimately reducing inflammation in distal organs (141, 142). This regulatory mechanism involving the “gut-brain axis” and “gut-lung axis” opens new perspectives for the application of APS.

### 4.5 Other activity

Mao et al. (143) discovered that Astragalus polysaccharide and its congener astragaloside IV significantly inhibit the expression of TGF-β1 and the phosphorylation of its downstream Smad2/3 proteins, thereby blocking the activation of myofibroblasts, reducing the excessive production and deposition of extracellular matrix components such as collagen and fibronectin, and consequently exerting an anti-hepatic fibrotic effect. Furthermore, Astragalus polysaccharide exhibits protective effects in various models of neurological damage. For example, in a diabetic rat model (144), APS was able to ameliorate learning and memory impairments and reduce neuronal death, a mechanism potentially related to the upregulation of phosphorylation levels of CREB, NMDA receptors, and CaMK II.

## 5 Cutting-edge applications and delivery innovations

### 5.1 The combination of Astragalus polysaccharide

The strategic combination of APS with conventional therapies represents a prominent trend, driven by the goals of achieving synergistic efficacy, attenuating side effects, and overcoming drug resistance. A key application is the enhancement of chemotherapy. Clinical research indicates that intravenous APS, when co-administered with paclitaxel and cisplatin, is applicable for treating advanced non-small cell lung cancer (145). A meta-analysis further confirmed that APS injection combined with the FOLFOX regimen significantly enhances therapeutic outcomes and mitigates adverse events in gastric cancer patients (146). Beyond clinical observations, the mechanistic basis for this synergy has been elucidated. For instance, APS potentiates the effect of cisplatin in ovarian cancer cells by activating the JNK pathway, which in turn regulates key apoptosis proteins like Bcl-2, Bax, and Caspase-3 (147). Furthermore, APS can counteract multidrug resistance MDR, a major cause of chemotherapeutic failure. Evidence suggests that APS re-sensitizes tumor cells by suppressing the expression of efflux pumps like P-glycoprotein, thereby increasing intracellular drug retention (148), In addition to enhancing efficacy, APS plays a critical role in mitigating chemotherapy-induced toxicity. Robust evidence from meta-analyses demonstrates that Astragalus-containing preparations significantly lower the risk of severe toxicities, including neutropenia, anemia, and gastrointestinal events (149–151). These systemic benefits are complemented by organ-specific protective effects. Research indicates that APS effectively attenuates anthracycline-induced cardiotoxicity, as evidenced by better preservation of cardiac function in patients (152, 153). The combinatory potential of APS extends beyond oncology into metabolic and inflammatory diseases. For example, a combination of Malus micromalus polysaccharide and APS ameliorated alcoholic fatty liver disease via modulation of the gut microbiota, while APS and matrine acted synergistically against ulcerative colitis (154, 155). Collectively, this evidence highlights the considerable potential of APS as a versatile “platform” therapeutic, capable of being integrated into multi-agent regimens to enhance efficacy and reduce toxicity across a spectrum of complex diseases.

### 5.2 Applications as a potential natural carrier material

Nanoparticle delivery systems have become a central focus in APS delivery research due to their advantages in protecting the drug, improving solubility, prolonging *in vivo* circulation time, and enabling targeted delivery. Poly (lactic-co-glycolic acid) (PLGA) is an FDA-approved biodegradable and biocompatible polymer widely used for drug delivery (156, 157). Researchers have successfully prepared PLGA nanoparticles loaded with APS (158). These nanoparticles can not only effectively encapsulate APS but also act as an immune adjuvant to enhance its immunogenicity (159). One study co-encapsulated APS and gold nanorods (AuNRs) into PLGA-polyethylene glycol (PEG) nanoparticles for combined photothermal-immunotherapy of breast cancer. The results showed that the encapsulation efficiency (EE) for APS could reach 54.89 ± 2.07% (160). Following local injection into the tumor, these nanoparticles effectively accumulated in the tumor tissue, exerting a synergistic therapeutic effect.

Liposomes, as a classic nanocarrier, are highly suitable for delivering polysaccharide-based drugs due to their cell membrane-like structure and excellent biocompatibility (161). Studies have shown that formulating APS into Astragalus polysaccharide liposomes (APS-Ls) can significantly improve its stability and delivery efficiency. One study successfully prepared APS-Ls with a particle size of less than 150 nm, achieving an entrapment rate of 47.50% ± 0.15% and a drug loading capacity of 5.40% ± 0.17% (162). Liposomes can protect APS from degradation by endogenous enzymes, prolong its circulation time, and potentially amplify its immunomodulatory effects by enhancing cellular uptake. Encapsulation efficiency (EE) and loading capacity (LC) are key metrics for evaluating the drug loading capability of nanocarriers. The EE of APS in PLGA and liposome systems can reach approximately 55% and 48%, respectively.

Employing APS as a self-assembling nanocarrier constitutes a remarkably innovative and fascinating recent research avenue. Research has found that APS molecules themselves possess the ability to self-assemble into nanoparticles (163). APS molecular chains contain numerous hydrophilic groups; however, under specific conditions, they can spontaneously aggregate via weak intermolecular interactions such as hydrogen bonding and van der Waals forces, forming nano-sized aggregates with internal hydrophobic cavities. This intrinsic property positions APS not merely as a bioactive drug substance, but also as a natural nanocarrier with exceptional biocompatibility (164).

### 5.3 Vaccine adjuvant

Vaccines represent the most effective public health tool for the prevention and control of infectious diseases. However, many modern vaccines, particularly subunit vaccines, recombinant protein vaccines, and nucleic acid vaccines, possess inherently weak immunogenicity and require the addition of an adjuvant to induce a sufficiently robust and durable protective immune response (165, 166). Adjuvants enhance, modulate, and prolong the host’s immune response to the antigen, thereby improving vaccine efficacy (167). Extensive research indicates that APS, when used as an adjuvant, can significantly enhance the immunogenicity of vaccines. For example, in various vaccine models (e.g., against H5N1 avian influenza, Newcastle disease virus, or ovalbumin), the addition of APS as an adjuvant consistently significantly elevated the titers of specific antibodies, including total IgG and key protective antibodies such as hemagglutination inhibition (HAI) antibodies (168–170). This indicates that APS can effectively promote the activation, proliferation, and differentiation of B cells into plasma cells, leading to abundant antibody production. Furthermore, APS can significantly promote the proliferative response of T lymphocytes, particularly splenocytes. In an influenza vaccine model, sera from the APS-adjuvanted group displayed elevated levels of both Th1 (e.g., IFN-γ, TNF-α) and Th2 (e.g., IL-4) cytokines, indicative of its capacity to elicit a balanced Th1/Th2 immune response. Comparative analyses revealed that APS adjuvantation was superior to either the vaccine alone or a standard aluminum adjuvant in enhancing HAI antibody titers and IgG levels in mice, and it significantly increased survival rates following viral infection. Notably, a low dosage of APS adjuvant demonstrated efficacy marginally superior to that of aluminum adjuvant, while concurrently inducing a more tempered inflammatory reaction (171). Collectively, these preclinical findings provide robust evidence endorsing APS as a potent and versatile vaccine adjuvant.

Mechanistic studies indicate that APS, via activation of the TLR4 signaling pathway, induces the secretion of Th1-polarizing cytokines (e.g., IL-12) alongside Th2-type cytokines (e.g., IL-4), facilitating a balanced Th1/Th2 hybrid immune response (172). This capacity for “bidirectional immunomodulation” constitutes a fundamental and distinguishing advantage of APS (173, 174).

Yakuboğulları et al. (175)utilized Astragalus polysaccharide (APS, gum tragacanth) and Astragaloside VII (AST-VII) to construct two nano-adjuvant carrier systems (APNS and ANS) and investigated their immunoenhancing effects as adjuvants for a seasonal influenza A (H3N2) vaccine. The study prepared nanoparticles via a pH-driven self-assembly method and evaluated the induced humoral immunity, cellular immune responses, and protective efficacy in a mouse model. The experimental results demonstrated that the APS-based system (APNS) primarily induced a Th2-biased immune response, characterized by a significant increase in vaccine-specific IgG1 antibody titers. In contrast, the composite nanoparticle integrating AST-VII with APS (ANS) guided a balanced Th1/Th2 immune response with mild Th17 activation, evidenced by the concurrent elevation of both IgG1 and IgG2a antibodies, promotion of IFN-γ and IL-17A secretion, and a remarkable enhancement of splenocyte proliferation. The underlying mechanism involves: APS, acting as both a carrier and immunomodulator, primarily drives the Th2 response by promoting B-cell activation and antibody production. Meanwhile, AST-VII, a triterpenoid saponin, synergizes with APS to balance T-cell differentiation, promoting the secretion of Th1-type (e.g., IFN-γ) and Th17-type (e.g., IL-17A) cytokines, thereby collectively shaping a balanced immune profile that combines strong antibody responses with robust cellular immunity.

Additionally, Yakuboğulları et al. (175, 176) systematically evaluated the immunoenhancing effects of Astragalus polysaccharide (APS) and Astragaloside VII (AST-VII) as nano-adjuvant carriers for both seasonal influenza A (H3N2) and Newcastle Disease Virus (NDV) vaccines. In these studies, nanoparticles were prepared via a pH-driven self-assembly method and administered to mouse models to assess humoral immunity, cellular immune responses, and protective efficacy. The results revealed distinct yet complementary roles for APS and AST-VII. The APS-based system (APNS) primarily induced a Th2-biased immune response, marked by a significant increase in vaccine-specific IgG1 antibody titers. In contrast, the composite nanoparticle integrating AST-VII with APS (ANS) guided a balanced Th1/Th2 immune response with mild Th17 activation, evidenced by the concurrent elevation of IgG1 and IgG2a antibodies, promotion of IFN-γ and IL-17A secretion, and enhanced splenocyte proliferation. Notably, in the NDV vaccine model, APS-based systems significantly boosted neutralizing antibody titers, outperforming a commercial oil-adjuvanted vaccine. Mechanistically, APS functions dually as both a delivery vehicle and an immunomodulator, primarily driving potent humoral immunity by enhancing B-cell responses. Meanwhile, AST-VII, a triterpenoid saponin, orchestrates T-cell responses by promoting balanced secretion of Th1- and Th2-related factors, thereby shaping an immune profile that effectively combines robust antibody production with cellular immunity.

This property enables APS-adjuvanted vaccines to stimulate not only high-titer neutralizing antibodies but also robust cytotoxic T lymphocyte (CTL) responses, offering a “dual-layered” protective strategy against challenging pathogens. A significant challenge, however, stems from the inherent nature of APS as a heterogeneous botanical extract; its chemical composition, molecular weight distribution, and purity are subject to variation based on the geographical source of the raw material and the specific extraction protocols employed, factors that directly influence its biological activity and safety profile. Therefore, the establishment of standardized manufacturing processes and rigorous quality control metrics is an essential prerequisite for the successful development of APS as a pharmaceutical-grade adjuvant. While TLR4 activation is implicated in its mechanism, the specific molecular entities within the complex APS mixture that are responsible for its core adjuvant activity, along with the complete intricate mechanism of its immunoregulatory network modulation, require further in-depth elucidation. Moreover, employing advanced techniques like systems biology and multi-omics approaches to comprehensively decipher its mode of action will be crucial for optimizing its application and potentially identifying novel biomarkers.

### 5.4 Applications in animal husbandry

Within the global context of pursuing green and sustainable development, the agriculture and animal husbandry sectors are experiencing a significant transformation. The escalating problem of antimicrobial resistance (AMR), driven by the misuse of antibiotics in animal feed, has prompted nations worldwide to implement policies restricting or banning their use as growth promoters. Consequently, identifying safe and efficacious antibiotic alternatives has emerged as a critical industry priority and an urgent necessity (23, 177). In light of increasingly stringent global regulations on in-feed antibiotics, the value of APS as a promising natural alternative has gained significant prominence (178). Studies (179) demonstrate that dietary supplementation with APS in cyclophosphamide (CPM)-treated broiler chickens enhances immune organ indices (e.g., spleen, thymus), stimulates immune cell proliferation and plasma nitric oxide synthase (NOS) activity, and significantly increases serum levels of immunoglobulins (IgG, IgA, and IgM). The gastrointestinal tract serves as the primary defense barrier for animal health. APS has been demonstrated to effectively ameliorate intestinal health (180). Its beneficial effects are multi-faceted: 1) APS optimizes gut morphology by increasing villus height and crypt depth, thereby expanding the absorptive surface area and enhancing the digestibility and absorption of nutrients; 2) It modulates the gut microbial community structure by inhibiting the growth of pathogenic bacteria and promoting the proliferation of beneficial bacteria (e.g., Lactobacillus and Bifidobacterium), thus maintaining a healthy microbial equilibrium; 3) It strengthens intestinal barrier integrity, reduces the absorption of endotoxins (e.g., LPS), and attenuates inflammatory responses (23, 181, 182).

## 6 Challenges and future perspectives

Despite notable advancements, the clinical translation of Astragalus polysaccharide (APS) is hindered by several interconnected challenges that define a clear roadmap for future research. These challenges can be grouped into two primary categories: the fundamental need for standardization and mechanistic validation, and the practical requirements for clinical translation.

The first category encompasses the need to overcome structural heterogeneity and establish causal mechanisms. A primary obstacle is the lack of standardization; future work must define critical quality attributes (e.g., molecular weight distribution, monosaccharide ratios) and establish a minimum data package for preclinical and clinical batches. To bridge the gap between structure and function, a prioritized research agenda should focus on: (1) validating direct receptor interactions using techniques like SPR/ITC with purified fractions and receptor ectodomains (e.g., TLR4/MD-2, MR); and (2) employing standardized functional assays in receptor-knockout models or with specific inhibitors to unequivocally attribute immunomodulatory effects. Underpinning these goals is the fundamental challenge of structural elucidation, which requires the application of advanced spectroscopy and AI to decipher structure-activity relationships.

The second category involves the translation of this mechanistic understanding into viable therapies. This requires a sophisticated PK/PD strategy using labeled fractions to track biodistribution and correlate structural features with exposure and immune readouts. From a manufacturing perspective, overcoming source variability is critical; this will likely involve synthetic biology and chemoenzymatic approaches to produce defined oligosaccharides, alongside navigating regulatory pathways for complex mixtures. Finally, clinical translation demands innovative trial designs with defined endpoints and biomarkers (e.g., cytokine panels, DC activation markers) for specific patient populations, such as in vaccine adjuvancy or chemo-immunotherapy.

Looking forward, research is poised to advance on the principles of precision and standardization. The integration of technologies like single-cell sequencing and spatial transcriptomics will provide a deeper, systems-level understanding of APS’s mechanisms. It is anticipated that through these concerted efforts, APS will evolve beyond its traditional origins into a novel class of glyco-pharmaceuticals with well-defined structures, playing a significant role in immunotherapy, chronic disease management, and the “One Health” paradigm.

## 7 Conclusion

Modern pharmacological investigations have uncovered the material basis for the diverse biological activities of Astragalus (Radix Astragali), a primary herb used for thousands of years in Traditional Chinese Medicine (TCM) to “tonify Qi.” Among its constituents, APS is widely acknowledged as one of its most significant and plentiful bioactive compounds. APS is not a single chemical entity but rather a heterogeneous class of complex biopolymers consisting of multiple monosaccharides connected via diverse glycosidic linkages. Our study provides a systematic dissection of the structural heterogeneity of APS and its immunoregulatory network, which operates as a multi-layered system with TLR4/MyD88 as its core, MR/NOD receptors as co-regulators, and T cell reprogramming as the effector mechanism. Through the application of multi-dimensional analytical techniques, we have elucidated the quantitative structure-activity relationship governing the interplay between its chemical structure, conformational dynamics, and receptor engagement. We have also systematically characterized the pleiotropic activities of APS extending beyond immunomodulation, positioning it as a “polyfunctional molecular weapon” with therapeutic potential against cancer, metabolic disorders, and inflammatory diseases.

## References

[1] Di Sotto A Vitalone A Di Giacomo S . Plant-derived nutraceuticals and immune system modulation: an evidence-based overview. Vaccines (Basel). (2020) 8:468. doi: 10.3390/vaccines8030468, PMID: 32842641 PMC7563161

[2] Yin M Zhang Y Li H . Advances in research on immunoregulation of macrophages by plant polysaccharides. Front Immunol. (2019) 10:145. doi: 10.3389/fimmu.2019.00145, PMID: 30804942 PMC6370632

[3] Zheng Y Ren W Zhang L Zhang Y Liu D Liu Y . A review of the pharmacological action of astragalus polysaccharide. Front Pharmacol. (2020) 11:349. doi: 10.3389/fphar.2020.00349, PMID: 32265719 PMC7105737

[4] Zhao LH Ma ZX Zhu J Yu XH Weng DP . Characterization of polysaccharide from Astragalus radix as the macrophage stimulator. Cell Immunol. (2011) 271:329–34. doi: 10.1016/j.cellimm.2011.07.011, PMID: 21937031

[5] Pu Y Zhu J Xu J Zhang S Bao Y . Antitumor effect of a polysaccharide from Pseudostellaria heterophylla through reversing tumor-associated macrophages phenotype. Int J Biol Macromol. (2022) 220:816–26. doi: 10.1016/j.ijbiomac.2022.08.111, PMID: 35988728

[6] Li WJ Tang XF Shuai XX Jiang CJ Liu X Wang LF . Mannose receptor mediates the immune response to ganoderma atrum polysaccharides in macrophages. J Agric Food Chem. (2017) 65:348–57. doi: 10.1021/acs.jafc.6b04888, PMID: 27931102

[7] Lin P Chen L Huang X Xiao F Fu L Jing D . Structural characteristics of polysaccharide GP2a in gardenia jasminoides and its immunomodulatory effect on macrophages. Int J Mol Sci. (2022) 23:11279. doi: 10.3390/ijms231911279, PMID: 36232580 PMC9569544

[8] Zhou L Liu Z Wang Z Yu S Long T Zhou X . Astragalus polysaccharides exerts immunomodulatory effects via TLR4-mediated MyD88-dependent signaling pathway *in vitro* and *in vivo*. Sci Rep. (2017) 7:44822. doi: 10.1038/srep44822, PMID: 28303957 PMC5355992

[9] Guo Y Wang L Liu K Li M Jin Y Gu L . A rapid and accurate UHPLC method for determination of monosaccharides in polysaccharides of different sources of radix astragali and its immune activity analysis. Molecules. (2024) 29:2287. doi: 10.3390/molecules29102287, PMID: 38792148 PMC11124152

[10] Lu J Chen X Zhang Y Xu J Zhang L Li Z . Astragalus polysaccharide induces anti-inflammatory effects dependent on AMPK activity in palmitate-treated RAW264.7 cells. Int J Mol Med. (2013) 31:1463–70. doi: 10.3892/ijmm.2013.1335, PMID: 23563695

[11] Wei J Dai Y Zhang N Wang Z Tian X Yan T . Natural plant-derived polysaccharides targeting macrophage polarization: a promising strategy for cancer immunotherapy. Front Immunol. (2024) 15:1408377. doi: 10.3389/fimmu.2024.1408377, PMID: 39351237 PMC11439661

[12] Li CX Liu Y Zhang YZ Li JC Lai J . Astragalus polysaccharide: a review of its immunomodulatory effect. Arch Pharm Res. (2022) 45:367–89. doi: 10.1007/s12272-022-01393-3, PMID: 35713852

[13] Sheng Z Liu J Yang B . Structure differences of water soluble polysaccharides in astragalus membranaceus induced by origin and their bioactivity. Foods. (2021) 10:1755. doi: 10.3390/foods10081755, PMID: 34441532 PMC8395020

[14] Blanco G Sánchez B Fdez-Riverola F Margolles A Lourenço A . In silico Approach for Unveiling the Glycoside Hydrolase Activities in Faecalibacterium prausnitzii Through a Systematic and Integrative Large-Scale Analysis. Front Microbiol. (2019) 10:517. doi: 10.3389/fmicb.2019.00517, PMID: 31024464 PMC6460054

[15] Zhang Y Li N Gong HX Zhao CJ Bao XR Liu W . Structural characterization and anti-tumor immunomodulatory effects of polysaccharides from Astragalus mongholicus with different cultivation modes. Int J Biol Macromol. (2025) 318:145233. doi: 10.1016/j.ijbiomac.2025.145233, PMID: 40516740

[16] Wu J Li C Bai L Wu J Bo R Ye M . Structural differences of polysaccharides from Astragalus before and after honey processing and their effects on colitis mice. Int J Biol Macromol. (2021) 182:815–24. doi: 10.1016/j.ijbiomac.2021.04.055, PMID: 33857512

[17] Liao J Li C Huang J Liu W Chen H Liao S . Structure characterization of honey-processed astragalus polysaccharides and its anti-inflammatory activity *in vitro*. Molecules. (2018) 23:168. doi: 10.3390/molecules23010168, PMID: 29342936 PMC6017495

[18] Tian Y Wu J Zheng Y Li M Xu X Chen H . Structural changes of polysaccharides from Astragulus after honey processing and their bioactivities on human gut microbiota. J Sci Food Agric. (2023) 103:7241–50. doi: 10.1002/jsfa.12808, PMID: 37358876

[19] Gu D Wang Y Jin H Kang S Liu Y Zan K . Changes of physicochemical properties and immunomodulatory activity of polysaccharides during processing of polygonum multiflorum thunb. Front Pharmacol. (2022) 13:934710. doi: 10.3389/fphar.2022.934710, PMID: 35784754 PMC9243645

[20] Shang H Wang M Li R Duan M Wu H Zhou H . Extraction condition optimization and effects of drying methods on physicochemical properties and antioxidant activities of polysaccharides from Astragalus cicer L. Sci Rep. (2018) 8:3359. doi: 10.1038/s41598-018-21295-z, PMID: 29463789 PMC5820361

[21] Shang HM Zhou HZ Li R Duan MY Wu HX Lou YJ . Extraction optimization and influences of drying methods on antioxidant activities of polysaccharide from cup plant (Silphium perfoliatum L.). PloS One. (2017) 12:e0183001. doi: 10.1371/journal.pone.0183001, PMID: 28837625 PMC5570291

[22] Chang X Chen X Guo Y Gong P Pei S Wang D . Advances in chemical composition, extraction techniques, analytical methods, and biological activity of astragali radix. Molecules. (2022) 27:1058. doi: 10.3390/molecules27031058, PMID: 35164321 PMC8839891

[23] Liang H Tao S Wang Y Zhao J Yan C Wu Y . Astragalus polysaccharide: implication for intestinal barrier, anti-inflammation, and animal production. Front Nutr. (2024) 11:1364739. doi: 10.3389/fnut.2024.1364739, PMID: 38757131 PMC11096541

[24] Fitzgerald KA Kagan JC . Toll-like receptors and the control of immunity. Cell. (2020) 180:1044–66. doi: 10.1016/j.cell.2020.02.041, PMID: 32164908 PMC9358771

[25] McAleer JP Vella AT . Understanding how lipopolysaccharide impacts CD4 T-cell immunity. Crit Rev Immunol. (2008) 28:281–99. doi: 10.1615/critrevimmunol.v28.i4.20, PMID: 19166381 PMC3549535

[26] Shimazu R Akashi S Ogata H Nagai Y Fukudome K Miyake K . MD-2, a molecule that confers lipopolysaccharide responsiveness on Toll-like receptor 4. J Exp Med. (1999) 189:1777–82. doi: 10.1084/jem.189.11.1777, PMID: 10359581 PMC2193086

[27] Cox E Verdonck F Vanrompay D Goddeeris B . Adjuvants modulating mucosal immune responses or directing systemic responses towards the mucosa. Vet Res. (2006) 37:511–39. doi: 10.1051/vetres:2006014, PMID: 16611561

[28] Rabinovich GA Croci DO . Regulatory circuits mediated by lectin-glycan interactions in autoimmunity and cancer. Immunity. (2012) 36:322–35. doi: 10.1016/j.immuni.2012.03.004, PMID: 22444630

[29] Sahasrabudhe NM Dokter-Fokkens J De Vos P . Particulate β-glucans synergistically activate TLR4 and Dectin-1 in human dendritic cells. Mol Nutr Food Res. (2016) 60:2514–22. doi: 10.1002/mnfr.201600356, PMID: 27358258

[30] Medzhitov R Preston-Hurlburt P Janeway CA Jr. A human homologue of the Drosophila Toll protein signals activation of adaptive immunity. Nature. (1997) 388:394–7. doi: 10.1038/41131, PMID: 9237759

[31] Poltorak A He X Smirnova I Liu MY Van Huffel C Du X . Defective LPS signaling in C3H/HeJ and C57BL/10ScCr mice: mutations in Tlr4 gene. Science. (1998) 282:2085–8. doi: 10.1126/science.282.5396.2085, PMID: 9851930

[32] Shao BM Xu W Dai H Tu P Li Z Gao XM . A study on the immune receptors for polysaccharides from the roots of Astragalus membranaceus, a Chinese medicinal herb. Biochem Biophys Res Commun. (2004) 320:1103–11. doi: 10.1016/j.bbrc.2004.06.065, PMID: 15249203

[33] Kong F Chen T Li X Jia Y . The current application and future prospects of astragalus polysaccharide combined with cancer immunotherapy: A review. Front Pharmacol. (2021) 12:737674. doi: 10.3389/fphar.2021.737674, PMID: 34721026 PMC8548714

[34] Li BX Li WY Tian YB Guo SX Huang YM Xu DN . Polysaccharide of atractylodes macrocephala koidz enhances cytokine secretion by stimulating the TLR4-myD88-NF-κB signaling pathway in the mouse spleen. J Med Food. (2019) 22:937–43. doi: 10.1089/jmf.2018.4393, PMID: 31448992

[35] Mann DL . The emerging role of innate immunity in the heart and vascular system: for whom the cell tolls. Circ Res. (2011) 108:1133–45. doi: 10.1161/circresaha.110.226936, PMID: 21527743 PMC3084988

[36] Faggioni R Fantuzzi G Fuller J Dinarello CA Feingold KR Grunfeld C . IL-1β mediates leptin induction during inflammation. Am J Physiology-Regul. Integr Comp Physiol. (1998) 274:R204–8. doi: 10.1152/ajpregu.1998.274.1.R204, PMID: 9458919

[37] Feng S Ding H Liu L Peng C Huang Y Zhong F . Astragalus polysaccharide enhances the immune function of RAW264.7 macrophages via the NF-κB p65/MAPK signaling pathway. Exp Ther Med. (2021) 21:20. doi: 10.3892/etm.2020.9452, PMID: 33235629 PMC7678613

[38] Wei W Xiao HT Bao WR Ma DL Leung CH Han XQ . TLR-4 may mediate signaling pathways of Astragalus polysaccharide RAP induced cytokine expression of RAW264.7 cells. J Ethnopharmacol. (2016) 179:243–52. doi: 10.1016/j.jep.2015.12.060, PMID: 26743224

[39] Wei Z Weng S Wang L Mao Z . Mechanism of Astragalus polysaccharides in attenuating insulin resistance in Rats with type 2 diabetes mellitus via the regulation of liver microRNA−203a−3p. Mol Med Rep. (2018) 17:1617–24. doi: 10.3892/mmr.2017.8084, PMID: 29257218 PMC5780102

[40] Feinberg H Jégouzo SAF Lasanajak Y Smith DF Drickamer K Weis WI . Structural analysis of carbohydrate binding by the macrophage mannose receptor CD206. J Biol Chem. (2021) 296:100368. doi: 10.1016/j.jbc.2021.100368, PMID: 33545173 PMC7949135

[41] Zlotnikov ID Kudryashova EV . Computer simulation of the receptor-ligand interactions of mannose receptor CD206 in comparison with the lectin concanavalin A model. Biochem (Mosc). (2022) 87:54–69. doi: 10.1134/s0006297922010059, PMID: 35491020 PMC8769089

[42] Taylor ME Drickamer K . Structural requirements for high affinity binding of complex ligands by the macrophage mannose receptor. J Biol Chem. (1993) 268:399–404. doi: 10.1016/S0021-9258(18)54164-8, PMID: 8416946

[43] Gazi U Martinez-Pomares L . Influence of the mannose receptor in host immune responses. Immunobiology. (2009) 214:554–61. doi: 10.1016/j.imbio.2008.11.004, PMID: 19162368

[44] Zamze S Martinez-Pomares L Jones H Taylor PR Stillion RJ Gordon S . Recognition of bacterial capsular polysaccharides and lipopolysaccharides by the macrophage mannose receptor. J Biol Chem. (2002) 277:41613–23. doi: 10.1074/jbc.M207057200, PMID: 12196537

[45] Stahl PD Ezekowitz RA . The mannose receptor is a pattern recognition receptor involved in host defense. Curr Opin Immunol. (1998) 10:50–5. doi: 10.1016/s0952-7915(98)80031-9, PMID: 9523111

[46] Li W Hu X Wang S Jiao Z Sun T Liu T . Characterization and anti-tumor bioactivity of astragalus polysaccharides by immunomodulation. Int J Biol Macromol. (2020) 145:985–97. doi: 10.1016/j.ijbiomac.2019.09.189, PMID: 31669273

[47] Adams EL Rice PJ Graves B Ensley HE Yu H Brown GD . Differential high-affinity interaction of dectin-1 with natural or synthetic glucans is dependent upon primary structure and is influenced by polymer chain length and side-chain branching. J Pharmacol Exp Ther. (2008) 325:115–23. doi: 10.1124/jpet.107.133124, PMID: 18171906

[48] Brown GD . Dectin-1: a signalling non-TLR pattern-recognition receptor. Nat Rev Immunol. (2006) 6:33–43. doi: 10.1038/nri1745, PMID: 16341139

[49] Brown GD Gordon S . Immune recognition. A new receptor for beta-glucans. Nature. (2001) 413:36–7. doi: 10.1038/35092620, PMID: 11544516

[50] Ariyoshi W Hara S Koga A Nagai-Yoshioka Y Yamasaki R . Biological effects of β-glucans on osteoclastogenesis. Molecules. (2021) 26:1982. doi: 10.3390/molecules26071982, PMID: 33915775 PMC8036280

[51] Manabe N Yamaguchi Y . 3D structural insights into β-glucans and their binding proteins. Int J Mol Sci. (2021) 22:1578. doi: 10.3390/ijms22041578, PMID: 33557270 PMC7915573

[52] Wen P Větvička V Crich D . Synthesis and evaluation of oligomeric thioether-linked carbacyclic β-(1→3)-glucan mimetics. J Org. Chem. (2019) 84:5554–63. doi: 10.1021/acs.joc.9b00504, PMID: 30933504 PMC6528479

[53] Meijerink M Rösch C Taverne N Venema K Gruppen H Schols HA . Structure dependent-immunomodulation by sugar beet arabinans via a SYK tyrosine kinase-dependent signaling pathway. Front Immunol. (2018) 9:1972. doi: 10.3389/fimmu.2018.01972, PMID: 30369923 PMC6194903

[54] Wagener M Hoving JC Ndlovu H Marakalala MJ . Dectin-1-syk-CARD9 signaling pathway in TB immunity. Front Immunol. (2018) 9:225. doi: 10.3389/fimmu.2018.00225, PMID: 29487599 PMC5816931

[55] De Marco Castro E Calder PC Roche HM . β-1,3/1,6-glucans and immunity: state of the art and future directions. Mol Nutr Food Res. (2021) 65:e1901071. doi: 10.1002/mnfr.201901071, PMID: 32223047 PMC7816268

[56] Goodridge HS Reyes CN Becker CA Katsumoto TR Ma J Wolf AJ . Activation of the innate immune receptor Dectin-1 upon formation of a 'phagocytic synapse'. Nature. (2011) 472:471–5. doi: 10.1038/nature10071, PMID: 21525931 PMC3084546

[57] Tian J Ma J Ma K Guo H Baidoo SE Zhang Y . β-Glucan enhances antitumor immune responses by regulating differentiation and function of monocytic myeloid-derived suppressor cells. Eur J Immunol. (2013) 43:1220–30. doi: 10.1002/eji.201242841, PMID: 23424024

[58] Ferwerda G Meyer-Wentrup F Kullberg BJ Netea MG Adema GJ . Dectin-1 synergizes with TLR2 and TLR4 for cytokine production in human primary monocytes and macrophages. Cell Microbiol. (2008) 10:2058–66. doi: 10.1111/j.1462-5822.2008.01188.x, PMID: 18549457

[59] Xi Y Liu R Zhang X Guo Q Zhang X Yang Z . A bibliometric analysis of metabolic reprogramming in the tumor microenvironment from 2003 to 2022. Cancer Rep (Hoboken). (2024) 7:e2146. doi: 10.1002/cnr2.2146, PMID: 39158178 PMC11331499

[60] Barber DL Wherry EJ Masopust D Zhu B Allison JP Sharpe AH . Restoring function in exhausted CD8 T cells during chronic viral infection. Nature. (2006) 439:682–7. doi: 10.1038/nature04444, PMID: 16382236

[61] Wherry EJ Kurachi M . Molecular and cellular insights into T cell exhaustion. Nat Rev Immunol. (2015) 15:486–99. doi: 10.1038/nri3862, PMID: 26205583 PMC4889009

[62] Shen J Zhang M Zhang K Qin Y Liu M Liang S . Effect of Angelica polysaccharide on mouse myeloid-derived suppressor cells. Front Immunol. (2022) 13:989230. doi: 10.3389/fimmu.2022.989230, PMID: 36159871 PMC9500156

[63] Xu J Zhang J Wang J . The application of traditional chinese medicine against the tumor immune escape. J Transl Int Med. (2020) 8:203–4. doi: 10.2478/jtim-2020-0032, PMID: 33511046 PMC7805287

[64] He Z Liu X Qin S Yang Q Na J Xue Z . Anticancer mechanism of astragalus polysaccharide and its application in cancer immunotherapy. Pharm (Basel). (2024) 17:636. doi: 10.3390/ph17050636, PMID: 38794206 PMC11124422

[65] Sun LI Zhuo S Li X Kong H Du W Zhou C . Astragalus polysaccharide enhances the therapeutic efficacy of cisplatin in triple-negative breast cancer through multiple mechanisms. Oncol Res. (2025) 33:641–51. doi: 10.32604/or.2024.050057, PMID: 40109863 PMC11915043

[66] Chang FL Tsai KC Lin TY Yang TW Lo YN Chen WC . Astragalus membranaceus-Derived Anti-Programmed Death-1 Monoclonal Antibodies with Immunomodulatory Therapeutic Effects against Tumors. BioMed Res Int. (2020) 2020:3415471. doi: 10.1155/2020/3415471, PMID: 32190660 PMC7073506

[67] Fontenot JD Gavin MA Rudensky AY . Foxp3 programs the development and function of CD4+CD25+ regulatory T cells. Nat Immunol. (2003) 4:330–6. doi: 10.1038/ni904, PMID: 12612578

[68] Li Q Bao JM Li XL Zhang T Shen XH . Inhibiting effect of Astragalus polysaccharides on the functions of CD4+CD25 highTreg cells in the tumor microenvironment of human hepatocellular carcinoma. Chin Med J (Engl). (2012) 125:786–93. doi: 10.3760/cma.j.issn.0366-6999.2012.05.012, PMID: 22490576

[69] Xu Q Cheng W Wei J Ou Y Xiao X Jia Y . Synergist for antitumor therapy: Astragalus polysaccharides acting on immune microenvironment. Discov Oncol. (2023) 14:179. doi: 10.1007/s12672-023-00798-w, PMID: 37741920 PMC10517906

[70] Lai X Xia W Wei J Ding X . Therapeutic effect of astragalus polysaccharides on hepatocellular carcinoma H22-bearing mice. Dose Respon. (2017) 15:1559325816685182. doi: 10.1177/1559325816685182, PMID: 28210201 PMC5298564

[71] Liu QY Yao YM Yu Y Dong N Sheng ZY . Astragalus polysaccharides attenuate postburn sepsis via inhibiting negative immunoregulation of CD4+ CD25(high) T cells. PloS One. (2011) 6:e19811. doi: 10.1371/journal.pone.0019811, PMID: 21698274 PMC3115936

[72] Zhao HM Wang Y Huang XY Huang MF Xu R Yue HY . Astragalus polysaccharide attenuates rat experimental colitis by inducing regulatory T cells in intestinal Peyer's patches. World J Gastroenterol. (2016) 22:3175–85. doi: 10.3748/wjg.v22.i11.3175, PMID: 27003994 PMC4789992

[73] Han Y Yu C Yu Y . Astragalus polysaccharide alleviates alveolar bone destruction by regulating local osteoclastogenesis during periodontitis. J Appl BioMed. (2021) 19:97–104. doi: 10.32725/jab.2021.010, PMID: 34907709

[74] Proell M Riedl SJ Fritz JH Rojas AM Schwarzenbacher R The Nod-like receptor (NLR) family: a tale of similarities and differences. PLoS One. (2008) 3:e2119. doi: 10.1371/journal.pone.0002119, PMID: 18446235 PMC2323615

[75] Zhan X Li Q Xu G Xiao X Bai Z The mechanism of NLRP3 inflammasome activation and its pharmacological inhibitors. Front Immunol. (2022) 13:1109938. doi: 10.3389/fimmu.2022.1109938, PMID: 36741414 PMC9889537

[76] Tian Z Liu Y Yang B Zhang J He H Ge H . Astagalus Polysaccharide Attenuates Murine Colitis through Inhibiton of the NLRP3 Inflammasome. Planta Med. (2017)83:70–77. doi: 10.1055/s-0042-108589, PMID: 27280937

[77] Xu J Zhang Q Li Z Gao Y Pang Z Wu Y . Astragalus Polysaccharides Attenuate Ovalbumin-Induced Allergic Rhinitis in Rats by Inhibiting NLRP3 Inflammasome Activation and NOD2-Mediated NF-κB Activation. J Med Food. (2021) 24:1–9. doi: 10.1089/jmf.2020.4750, PMID: 33370169

[78] Li K Li S Du Y Qin X . Screening and structure study of active components of Astragalus polysaccharide for injection based on different molecular weights. J Chromatogr B Analyt Technol BioMed Life Sci. (2020) 1152:122255. doi: 10.1016/j.jchromb.2020.122255, PMID: 32673831

[79] Li K Li S Wang D Li X Wu X Liu X . Extraction, characterization, antitumor and immunological activities of hemicellulose polysaccharide from astragalus radix herb residue. Molecules. (2019) 24:3644. doi: 10.3390/molecules24203644, PMID: 31601012 PMC6833037

[80] Lim JD Yu CY Kim SH Chung IM . Structural characterization of an intestinal immune system-modulating arabino-3,6-galactan-like polysaccharide from the above-ground part of Astragalus membranaceus (Bunge). Carbohydr Polym. (2016) 136:1265–72. doi: 10.1016/j.carbpol.2015.10.029, PMID: 26572470

[81] Lv X Chen D Yang L Zhu N Li J Zhao J . Comparative studies on the immunoregulatory effects of three polysaccharides using high content imaging system. Int J Biol Macromol. (2016) 86:28–42. doi: 10.1016/j.ijbiomac.2016.01.048, PMID: 26783639

[82] Niu Y Wang H Xie Z Whent M Gao X Zhang X . Structural analysis and bioactivity of a polysaccharide from the roots of Astragalus membranaceus (Fisch) Bge. var. mongolicus (Bge.) Hsiao. Food Chem. (2011) 128:620–6. doi: 10.1016/j.foodchem.2011.03.055

[83] Li C Talapphet N Palanisamy S Ma N Cho ML You S . The relationship between structural properties and activation of RAW264.7 and natural killer (NK) cells by sulfated polysaccharides extracted from Astragalus membranaceus roots. Proc. Biochem. (2020) 97:140–8. doi: 10.1016/j.procbio.2020.06.021

[84] Yin JY Chan BC Yu H Lau IY Han XQ Cheng SW . Separation, structure characterization, conformation and immunomodulating effect of a hyperbranched heteroglycan from Radix Astragali. Carbohydr Polym. (2012) 87:667–75. doi: 10.1016/j.carbpol.2011.08.045, PMID: 34663019

[85] Liu AJ Yu J Ji HY Zhang HC Zhang Y Liu HP . Extraction of a novel cold-water-soluble polysaccharide from astragalus membranaceus and its antitumor and immunological activities. Molecules. (2017) 23. doi: 10.3390/molecules23010062, PMID: 29283407 PMC6017583

[86] Yu J Ji HY Liu AJ . Alcohol-soluble polysaccharide from Astragalus membranaceus: Preparation, characteristics and antitumor activity. Int J Biol Macromol. (2018) 118:2057–64. doi: 10.1016/j.ijbiomac.2018.07.073, PMID: 30009907

[87] Li K Cui LJ Cao YX Li SY Shi LX Qin XM . UHPLC Q-exactive MS-based serum metabolomics to explore the effect mechanisms of immunological activity of astragalus polysaccharides with different molecular weights. Front Pharmacol. (2020) 11:595692. doi: 10.3389/fphar.2020.595692, PMID: 33390982 PMC7774101

[88] Wang K Zhou Y Li M Chen Z Wu Z Ji W . Structural elucidation and immunomodulatory activities *in vitro* of type I and II arabinogalactans from different origins of Astragalus membranaceus. Carbohydr. Polym. (2024) 333:121974. doi: 10.1016/j.carbpol.2024.121974, PMID: 38494227

[89] Hu L Sun Q Liu Z Huang H Zhao E Chen H . Structural characterization of APSN from astragalus membranaceus and its potential therapeutic effect on immune dysregulation and tissue damage. J Agric Food Chem. (2025) 73:4042–54. doi: 10.1021/acs.jafc.4c08632, PMID: 39918058

[90] Li K Cao YX Jiao SM Du GH Du YG Qin XM . Structural characterization and immune activity screening of polysaccharides with different molecular weights from astragali radix. Front Pharmacol. (2020) 11:582091. doi: 10.3389/fphar.2020.582091, PMID: 33390949 PMC7774520

[91] Di Lorenzo F Silipo A Molinaro A Parrilli M Schiraldi C D'Agostino A . The polysaccharide and low molecular weight components of Opuntia ficus indica cladodes: Structure and skin repairing properties. Carbohydr Polym. (2017) 157:128–36. doi: 10.1016/j.carbpol.2016.09.073, PMID: 27987833

[92] Ren L Wang X Li S Li J Zhu X Zhang L . Effect of gamma irradiation on structure, physicochemical and immunomodulatory properties of Astragalus polysaccharides. Int J Biol Macromol. (2018) 120:641–9. doi: 10.1016/j.ijbiomac.2018.08.138, PMID: 30171942

[93] Sun L Wang C Shi Q Ma C . Preparation of different molecular weight polysaccharides from Porphyridium cruentum and their antioxidant activities. Int J Biol Macromol. (2009) 45:42–7. doi: 10.1016/j.ijbiomac.2009.03.013, PMID: 19447258

[94] Yu Y Shen M Song Q Xie J . Biological activities and pharmaceutical applications of polysaccharide from natural resources: A review. Carbohydr Polym. (2018) 183:91–101. doi: 10.1016/j.carbpol.2017.12.009, PMID: 29352896

[95] Roszczyk A Turło J Zagożdżon R Kaleta B . Immunomodulatory properties of polysaccharides from lentinula edodes. Int J Mol Sci. (2022) 23. doi: 10.3390/ijms23168980, PMID: 36012249 PMC9409024

[96] You R Wang K Liu J Liu M Luo L Zhang Y . A comparison study between different molecular weight polysaccharides derived from Lentinus edodes and their antioxidant activities *in vivo*. Pharm Biol. (2011) 49:1298–305. doi: 10.3109/13880209.2011.621960, PMID: 22077165

[97] Zhang Y Liu W Xu C Huang W He P . Characterization and antiproliferative effect of novel acid polysaccharides from the spent substrate of shiitake culinary-medicinal mushroom lentinus edodes (Agaricomycetes) cultivation. Int J Med Mushrooms. (2017) 19:395–403. doi: 10.1615/IntJMedMushrooms.v19.i5.20, PMID: 28845769

[98] Jeff IB Fan E Tian M Song C Yan J Zhou Y . *In vivo* anticancer and immunomodulating activities of mannogalactoglucan-type polysaccharides from Lentinus edodes (Berkeley) Singer. Cent. Eur J Immunol. (2016) 41:47–53. doi: 10.5114/ceji.2015.56962, PMID: 27095922 PMC4829809

[99] Lee JY Kim JY Lee YG Rhee MH Hong EK Cho JY . Molecular mechanism of macrophage activation by Exopolysaccharides from liquid culture of Lentinus edodes. J Microbiol Biotechnol. (2008) 18:355–64. doi: 10.1007/s00253-008-1439-9, PMID: 18309284

[100] Jiang Y Qi X Gao K Liu W Li N Cheng N . Relationship between molecular weight, monosaccharide composition and immunobiologic activity of Astragalus polysaccharides. Glycoconj. J. (2016) 33:755–61. doi: 10.1007/s10719-016-9669-z, PMID: 27129881

[101] Meng LZ Xie J Lv GP Hu DJ Zhao J Duan JA . A comparative study on immunomodulatory activity of polysaccharides from two official species of Ganoderma (Lingzhi). Nutr Cancer. (2014) 66:1124–31. doi: 10.1080/01635581.2014.948215, PMID: 25204488

[102] Bohn JA BeMiller JN . (1→ 3)-β-d-Glucans as biological response modifiers: a review of structure-functional activity relationships.Carbohydr Polymers. (1995) 28:3–14. doi: 10.1016/0144-8617(95)00076-3

[103] Li K Li XQ Li GX Cui LJ Qin XM Li ZY . Relationship between the structure and immune activity of components from the active polysaccharides APS-II of astragali radix by enzymolysis of endo α-1,4-glucanase. Front Pharmacol. (2022) 13:839635. doi: 10.3389/fphar.2022.839635, PMID: 35281923 PMC8913491

[104] Wang Y Zhao Y Zhang Q Qiao S Qi C Zhang Y . Isolation and structure elucidation of novel glucan from Astragalus mongholicus. Zhongcaoyao. (1994) 2001:962–4.

[105] Araújo-Rodrigues H Sousa AS Relvas JB Tavaria FK Pintado M . An overview on mushroom polysaccharides: health-promoting properties, prebiotic and gut microbiota modulation effects and structure-function correlation. Carbohydr. Polym. (2024) 333:121978. doi: 10.1016/j.carbpol.2024.121978, PMID: 38494231

[106] Bender DA Cunningham SM . Introduction to nutrition and metabolism. Boca Raton: CRC Press (2021).

[107] Guo MQ Hu X Wang C Ai L . Polysaccharides: structure and solubility. Solubil. Polysaccharides. (2017) 2:8–21. doi: 10.5772/intechopen.71570Provisionalchapter

[108] Han B Baruah K Cox E Vanrompay D Bossier P . Structure-functional activity relationship of β-glucans from the perspective of immunomodulation: A mini-review. Front Immunol. (2020) 11:658. doi: 10.3389/fimmu.2020.00658, PMID: 32391005 PMC7188827

[109] Liu J Zhang J Feng J Tang C Yan M Zhou S . Multiple fingerprint-activity relationship assessment of immunomodulatory polysaccharides from ganoderma lucidum based on chemometric methods. Molecules. (2023) 28. doi: 10.3390/molecules28072913, PMID: 37049676 PMC10096448

[110] Hounsell EF Davies MJ . Role of protein glycosylation in immune regulation. Ann Rheum. Dis. (1993) 52:S22–9. doi: 10.1136/ard.52.suppl_1.s22, PMID: 8481055 PMC1035023

[111] Guo ZZ Lou YM Kong MY Luo Q Liu ZQ Wu JJ . A systematic review of phytochemistry, pharmacology and pharmacokinetics on astragali radix: implications for astragali radix as a personalized medicine. Int J Mol Sci. (2019) 20. doi: 10.3390/ijms20061463, PMID: 30909474 PMC6470777

[112] Zhang R Qin X Zhang T Li Q Zhang J Zhao J . Astragalus polysaccharide improves insulin sensitivity via AMPK activation in 3T3-L1 adipocytes. Molecules. (2018) 23:2711. doi: 10.3390/molecules23102711, PMID: 30347867 PMC6222405

[113] Abel ED Peroni O Kim JK Kim YB Boss O Hadro E . Adipose-selective targeting of the GLUT4 gene impairs insulin action in muscle and liver. Nature. (2001) 409:729–33. doi: 10.1038/35055575, PMID: 11217863

[114] Liu M Wu K Mao X Wu Y Ouyang J . Astragalus polysaccharide improves insulin sensitivity in KKAy mice: regulation of PKB/GLUT4 signaling in skeletal muscle. J Ethnopharmacol. (2010) 127:32–7. doi: 10.1016/j.jep.2009.09.055, PMID: 19800959

[115] Zhao M Zhang ZF Ding Y Wang JB Li Y . Astragalus polysaccharide improves palmitate-induced insulin resistance by inhibiting PTP1B and NF-κB in C2C12 myotubes. Molecules. (2012) 17:7083–92. doi: 10.3390/molecules17067083, PMID: 22728372 PMC6268810

[116] Gu C Zeng Y Tang Z Wang C He Y Feng X . Astragalus polysaccharides affect insulin resistance by regulating the hepatic SIRT1-PGC-1α/PPARα-FGF21 signaling pathway in male Sprague Dawley rats undergoing catch-up growth. Mol Med Rep. (2015) 12:6451–60. doi: 10.3892/mmr.2015.4245, PMID: 26323321 PMC4626146

[117] Zhang J Feng Q . Pharmacological effects and molecular protective mechanisms of astragalus polysaccharides on nonalcoholic fatty liver disease. Front Pharmacol. (2022) 13:854674. doi: 10.3389/fphar.2022.854674, PMID: 35308224 PMC8929346

[118] Yue X Hao W Wang M Fu Y . Astragalus polysaccharide ameliorates insulin resistance in HepG2 cells through activating the STAT5/IGF-1 pathway. Immun Inflammation Dis. (2023) 11:e1071. doi: 10.1002/iid3.1071, PMID: 38018587 PMC10664394

[119] Liu H Bai J Weng X Wang T Li M . Amelioration of insulin resistance in rat cells by Astragalus polysaccharides and associated mechanisms. Exp Ther Med. (2014) 7:1599–604. doi: 10.3892/etm.2014.1626, PMID: 24926351 PMC4043571

[120] Younossi Z Anstee QM Marietti M Hardy T Henry L Eslam M . Global burden of NAFLD and NASH: trends, predictions, risk factors and prevention. Nat Rev Gastroenterol Hepatol. (2018) 15:11–20. doi: 10.1038/nrgastro.2017.109, PMID: 28930295

[121] Rivera-Esteban J Armandi A Augustin S Bugianesi E . Outcomes and potential surrogate markers for future clinical trials of non-alcoholic steatohepatitis cirrhosis. Liver. Int. (2021) 41:1999–2008. doi: 10.1111/liv.15013, PMID: 34242466 PMC8457215

[122] Xu Y Xu C Huang J Xu C Xiong Y . Astragalus polysaccharide attenuates diabetic nephropathy by reducing apoptosis and enhancing autophagy through activation of Sirt1/FoxO1 pathway. Int Urol. Nephrol. (2024) 56:3067–78. doi: 10.1007/s11255-024-04038-0, PMID: 38653852

[123] Dong M Li J Yang D Li M Wei J . Biosynthesis and pharmacological activities of flavonoids, triterpene saponins and polysaccharides derived from astragalus membranaceus. Molecules. (2023) 28:5018. doi: 10.3390/molecules28135018, PMID: 37446680 PMC10343288

[124] Li W Hu X Li Y Song K . Cytotoxicity and growth-inhibiting activity of Astragalus polysaccharides against breast cancer via the regulation of EGFR and ANXA1. J Nat Med. (2021) 75:854–70. doi: 10.1007/s11418-021-01525-x, PMID: 34043154

[125] Wu CY Ke Y Zeng YF Zhang YW Yu HJ . Anticancer activity of Astragalus polysaccharide in human non-small cell lung cancer cells. Cancer Cell Int. (2017) 17:115. doi: 10.1186/s12935-017-0487-6, PMID: 29225515 PMC5716001

[126] Yang S Sun S Xu W Yu B Wang G Wang H . Astragalus polysaccharide inhibits breast cancer cell migration and invasion by regulating epithelial−mesenchymal transition via the Wnt/β−catenin signaling pathway. Mol Med Rep. (2020) 21:1819–32. doi: 10.3892/mmr.2020.10983, PMID: 32319619 PMC7057808

[127] Liu C Li H Wang K Zhuang J Chu F Gao C . Identifying the antiproliferative effect of astragalus polysaccharides on breast cancer: coupling network pharmacology with targetable screening from the cancer genome atlas. Front Oncol. (2019) 9:368. doi: 10.3389/fonc.2019.00368, PMID: 31157164 PMC6533882

[128] Shen WC Chen SC Wang CH Hung CM Peng MT Liu CT . Astragalus polysaccharides improve adjuvant chemotherapy-induced fatigue for patients with early breast cancer. Sci Rep. (2024) 14:25690. doi: 10.1038/s41598-024-76627-z, PMID: 39465324 PMC11514294

[129] Wang W Zhou H Sen A Zhang P Yuan L Zhou S . Recent advances in the mechanisms and applications of astragalus polysaccharides in liver cancer treatment: an overview. Molecules. (2025) 30:2792. doi: 10.3390/molecules30132792, PMID: 40649307 PMC12250682

[130] Wei W Li ZP Bian ZX Han QB . Astragalus polysaccharide RAP induces macrophage phenotype polarization to M1 via the notch signaling pathway. Molecules. (2019) 24:2016. doi: 10.3390/molecules24102016, PMID: 31137782 PMC6572696

[131] Zhao L Zhong Y Liang J Gao H Tang N . Effect of astragalus polysaccharide on the expression of VEGF and EGFR in mice with lewis transplantable lung cancer. J Coll Phys. Surg Pak. (2019) 29:392–4. doi: 10.29271/jcpsp.2019.04.392, PMID: 30925971

[132] Tian QE Li HD Yan M Cai HL Tan QY Zhang WY . Astragalus polysaccharides can regulate cytokine and P-glycoprotein expression in H22 tumor-bearing mice. World J Gastroenterol. (2012) 18:7079–86. doi: 10.3748/wjg.v18.i47.7079, PMID: 23323011 PMC3531697

[133] Tian QE De Li H Yan M Cai HL Tan QY Zhang WY . Effects of Astragalus polysaccharides on P-glycoprotein efflux pump function and protein expression in H22 hepatoma cells *in vitro*. BMC Comple. Altern Med. (2012) 12:94. doi: 10.1186/1472-6882-12-94, PMID: 22784390 PMC3493361

[134] Chen R Li Y Zuo L Xiong H Sun R Song X . Astragalus polysaccharides inhibits tumor proliferation and enhances cisplatin sensitivity in bladder cancer by regulating the PI3K/AKT/FoxO1 axis. Int J Biol Macromol. (2025) 311:143739. doi: 10.1016/j.ijbiomac.2025.143739, PMID: 40318719

[135] Li S Chen X Shi H Yi M Xiong B Li T . Tailoring traditional Chinese medicine in cancer therapy. Mol Cancer. (2025) 24:27. doi: 10.1186/s12943-024-02213-6, PMID: 39838407 PMC11749133

[136] Fu J Wang Z Huang L Zheng S Wang D Chen S . Review of the botanical characteristics, phytochemistry, and pharmacology of Astragalus membranaceus (Huangqi). Phytother Res. (2014) 28:1275–83. doi: 10.1002/ptr.5188, PMID: 25087616

[137] Dong N Li X Xue C Zhang L Wang C Xu X . Astragalus polysaccharides alleviates LPS-induced inflammation via the NF-κB/MAPK signaling pathway. J Cell Physiol. (2020) 235:5525–40. doi: 10.1002/jcp.29452, PMID: 32037545

[138] Liu T Zhang M Niu H Liu J Ruilian M Wang Y . Astragalus polysaccharide from Astragalus Melittin ameliorates inflammation via suppressing the activation of TLR-4/NF-κB p65 signal pathway and protects mice from CVB3-induced virus myocarditis. Int J Biol Macromol. (2019) 126:179–86. doi: 10.1016/j.ijbiomac.2018.12.207, PMID: 30586589

[139] Li D Liu Y Xu R Jia X Li X Huo C . Astragalus polysaccharide alleviates H(2)O(2)-triggered oxidative injury in human umbilical vein endothelial cells via promoting KLF2. Artif Cells Nanomed. Biotechnol. (2019) 47:2188–95. doi: 10.1080/21691401.2019.1621886, PMID: 31159593

[140] Zhang Y Lin X Xia L Xiong S Xia B Xie J . Progress on the anti-inflammatory activity and structure-efficacy relationship of polysaccharides from medical and edible homologous traditional chinese medicines. Molecules. (2024) 29:3852. doi: 10.3390/molecules29163852, PMID: 39202931 PMC11356930

[141] Chen L Song K . Astragalus polysaccharide in digestive health: A review of mechanisms, applications, and therapeutic potential. ChemSci. Adv. (2024) 01:209–25. doi: 10.69626/csa.2024.0209

[142] Zhang Y Ji W Qin H Chen Z Zhou Y Zhou Z . Astragalus polysaccharides alleviate DSS-induced ulcerative colitis in mice by restoring SCFA production and regulating Th17/Treg cell homeostasis in a microbiota-dependent manner. Carbohydr. Polym. (2025) 349:122829. doi: 10.1016/j.carbpol.2024.122829, PMID: 39643403

[143] Mao Q Chen C Liang H Zhong S Cheng X Li L . Astragaloside IV inhibits excessive mesangial cell proliferation and renal fibrosis caused by diabetic nephropathy via modulation of the TGF-β1/Smad/miR-192 signaling pathway. Exp Ther Med. (2019) 18:3053–61. doi: 10.3892/etm.2019.7887, PMID: 31572545 PMC6755437

[144] Zhang G Fang H Li Y Xu J Zhang D Sun Y . Neuroprotective effect of astragalus polysacharin on streptozotocin (STZ)-induced diabetic rats. Med Sci Monit. (2019) 25:135–41. doi: 10.12659/msm.912213, PMID: 30610831 PMC6330021

[145] Guo L Bai SP Zhao L Wang XH . Astragalus polysaccharide injection integrated with vinorelbine and cisplatin for patients with advanced non-small cell lung cancer: effects on quality of life and survival. Med Oncol. (2012) 29:1656–62. doi: 10.1007/s12032-011-0068-9, PMID: 21928106

[146] Zhang D Zheng J Ni M Wu J Wang K Duan X . Comparative efficacy and safety of Chinese herbal injections combined with the FOLFOX regimen for treating gastric cancer in China: a network meta-analysis. Oncotarget. (2017) 8:68873–89. doi: 10.18632/oncotarget.20320, PMID: 28978164 PMC5620304

[147] Li C Hong L Liu C Min J Hu M Guo W . Astragalus polysaccharides increase the sensitivity of SKOV3 cells to cisplatin. Arch Gynecol. Obstet. (2018) 297:381–6. doi: 10.1007/s00404-017-4580-9, PMID: 29103194

[148] Tian Q-E Yan M Cai H-L Tan Q-Y Zhang W-Y . Astragalus polysaccharides can regulate cytokine and P-glycoprotein expression in H22 tumor-bearing mice. World J Gastroenterol.: WJG. (2012) 18:7079. doi: 10.3748/wjg.v18.i47.7079, PMID: 23323011 PMC3531697

[149] Cheng M Hu J Zhao Y Jiang J Qi R Chen S . Efficacy and safety of astragalus-containing traditional chinese medicine combined with platinum-based chemotherapy in advanced gastric cancer: A systematic review and meta-analysis. Front Oncol. (2021) 11:632168. doi: 10.3389/fonc.2021.632168, PMID: 34422628 PMC8371531

[150] Lin S An X Guo Y Gu J Xie T Wu Q . Meta-analysis of astragalus-containing traditional chinese medicine combined with chemotherapy for colorectal cancer: efficacy and safety to tumor response. Front Oncol. (2019) 9:749. doi: 10.3389/fonc.2019.00749, PMID: 31456940 PMC6700271

[151] McCulloch M See C Shu XJ Broffman M Kramer A Fan WY . Astragalus-based Chinese herbs and platinum-based chemotherapy for advanced non-small-cell lung cancer: meta-analysis of randomized trials. J Clin Oncol. (2006) 24:419–30. doi: 10.1200/jco.2005.03.6392, PMID: 16421421

[152] Cao Y Ruan Y Shen T Huang X Li M Yu W . Astragalus polysaccharide suppresses doxorubicin-induced cardiotoxicity by regulating the PI3k/Akt and p38MAPK pathways. Oxid Med Cell Longev. (2014) 2014:674219. doi: 10.1155/2014/674219, PMID: 25386226 PMC4216718

[153] Cao Y Shen T Huang X Lin Y Chen B Pang J . Astragalus polysaccharide restores autophagic flux and improves cardiomyocyte function in doxorubicin-induced cardiotoxicity. Oncotarget. (2017) 8:4837–48. doi: 10.18632/oncotarget.13596, PMID: 27902477 PMC5341749

[154] Liu J Kong L Shao M Sun C Li C Wang Y . Seabuckthorn polysaccharide combined with astragalus polysaccharide ameliorate alcoholic fatty liver by regulating intestinal flora. Front Endocrinol (Lausanne). (2022) 13:1018557. doi: 10.3389/fendo.2022.1018557, PMID: 36246879 PMC9559367

[155] Yan X Lu QG Zeng L Li XH Liu Y Du XF . Synergistic protection of astragalus polysaccharides and matrine against ulcerative colitis and associated lung injury in rats. World J Gastroenterol. (2020) 26:55–69. doi: 10.3748/wjg.v26.i1.55, PMID: 31933514 PMC6952295

[156] Fu Y Kao WJ . Drug release kinetics and transport mechanisms of non-degradable and degradable polymeric delivery systems. Expert Opin Drug Delivery. (2010) 7:429–44. doi: 10.1517/17425241003602259, PMID: 20331353 PMC2846103

[157] Makadia HK Siegel SJ . Poly lactic-co-glycolic acid (PLGA) as biodegradable controlled drug delivery carrier. Polymers. (Basel). (2011) 3:1377–97. doi: 10.3390/polym3031377, PMID: 22577513 PMC3347861

[158] Xu S Wusiman A Liu Z Gu P Ni H Zhang Y . pH-responsive Astragalus polysaccharides-loaded poly(lactic-co-glycolic acid) nanoparticles and their *in vitro* immunogenicity. Int J Biol Macromol. (2019) 125:865–75. doi: 10.1016/j.ijbiomac.2018.12.156, PMID: 30576729

[159] Li N Zhang Y Han M Liu T Wu J Xiong Y . Self-adjuvant Astragalus polysaccharide-based nanovaccines for enhanced tumor immunotherapy: a novel delivery system candidate for tumor vaccines. Sci China Life Sci. (2024) 67:680–97. doi: 10.1007/s11427-023-2465-x, PMID: 38206438

[160] Xiong J Jiang B Luo Y Zou J Gao X Xu D . Multifunctional nanoparticles encapsulating astragalus polysaccharide and gold nanorods in combination with focused ultrasound for the treatment of breast cancer. Int J Nanomed. (2020) 15:4151–69. doi: 10.2147/ijn.S246447, PMID: 32606670 PMC7305853

[161] Chattopadhyay S Chen JY Chen HW Hu CJ . Nanoparticle vaccines adopting virus-like features for enhanced immune potentiation. Nanotheranostics. (2017) 1:244–60. doi: 10.7150/ntno.19796, PMID: 29071191 PMC5646730

[162] Fan YP WD Hu YL . Reparation condition optimization of astragalus polysaccharide liposome by orthogonal test. Chin Tradi. Herbal Drugs. (2011) 3:470–3.

[163] Yang B Wu X Zeng J Song J Qi T Yang Y . A multi-component nano-co-delivery system utilizing astragalus polysaccharides as carriers for improving biopharmaceutical properties of astragalus flavonoids. Int J Nanomed. (2023) 18:6705–24. doi: 10.2147/ijn.S434196, PMID: 38026532 PMC10656867

[164] Granata G Paterniti I Geraci C Cunsolo F Esposito E Cordaro M . Potential eye drop based on a calix[4]arene nanoassembly for curcumin delivery: enhanced drug solubility, stability, and anti-inflammatory effect. Mol Pharm. (2017) 14:1610–22. doi: 10.1021/acs.molpharmaceut.6b01066, PMID: 28394618

[165] Cui Y Ho M Hu Y Shi Y . Vaccine adjuvants: current status, research and development, licensing, and future opportunities. J Mater Chem B. (2024) 12:4118–37. doi: 10.1039/d3tb02861e, PMID: 38591323 PMC11180427

[166] Didierlaurent AM Laupèze B Di Pasquale A Hergli N Collignon C Garçon N . Adjuvant system AS01: helping to overcome the challenges of modern vaccines. Expert Rev Vaccines. (2017) 16:55–63. doi: 10.1080/14760584.2016.1213632, PMID: 27448771

[167] Coffman RL Sher A Seder RA . Vaccine adjuvants: putting innate immunity to work. Immunity. (2010) 33:492–503. doi: 10.1016/j.immuni.2010.10.002, PMID: 21029960 PMC3420356

[168] Abdullahi AY Kallon S Yu X Zhang Y Li G . Vaccination with astragalus and ginseng polysaccharides improves immune response of chickens against H5N1 avian influenza virus. BioMed Res Int. (2016) 2016:1510264. doi: 10.1155/2016/1510264, PMID: 27597953 PMC5002477

[169] Kong XF Hu YL Yin YL Wu GY Rui R Wang DY . Chinese herbal ingredients are effective immune stimulators for chickens infected with the Newcastle disease virus. Poult Sci. (2006) 85:2169–75. doi: 10.1093/ps/85.12.2169, PMID: 17135673

[170] Zhou Y Zong Y Liu Z Zhao H Zhao X Wang J . Astragalus polysaccharides enhance the immune response to OVA antigen in BALB/c mice. BioMed Res Int. (2021) 2021:9976079. doi: 10.1155/2021/9976079, PMID: 34258286 PMC8260300

[171] Zhao D Chen X Wang L Zhang J Zhao Z Yue N . Bidirectional and persistent immunomodulation of Astragalus polysaccharide as an adjuvant of influenza and recombinant SARS-CoV-2 vaccine. Int J Biol Macromol. (2023) 234:123635. doi: 10.1016/j.ijbiomac.2023.123635, PMID: 36801224 PMC9932796

[172] Wei W . Immunomodulating effects of natural polysaccharides isolated from astragali radix and dendrobii officinalis caulis. (2016).

[173] Wan X Yin Y Zhou C Hou L Cui Q Zhang X . Polysaccharides derived from Chinese medicinal herbs: A promising choice of vaccine adjuvants. Carbohydr Polym. (2022) 276:118739. doi: 10.1016/j.carbpol.2021.118739, PMID: 34823775

[174] Wang D Liu Y Zhao W . The adjuvant effects on vaccine and the immunomodulatory mechanisms of polysaccharides from traditional chinese medicine. Front Mol Biosci. (2021) 8:655570. doi: 10.3389/fmolb.2021.655570, PMID: 33869288 PMC8047473

[175] Yakuboğulları N Genç R Çöven F Nalbantsoy A Bedir E . Development of adjuvant nanocarrier systems for seasonal influenza A (H3N2) vaccine based on Astragaloside VII and gum tragacanth (APS). Vaccine. (2019) 37:3638–45. doi: 10.1016/j.vaccine.2019.05.038, PMID: 31155418

[176] Yakubogullari N Coven FO Cebi N Coven F Coven N Genc R . Evaluation of adjuvant activity of Astragaloside VII and its combination with different immunostimulating agents in Newcastle Disease vaccine. Biologicals. (2021) 70:28–37. doi: 10.1016/j.biologicals.2021.01.005, PMID: 33608170

[177] Wu S . Effect of dietary Astragalus membranaceus polysaccharide on the growth performance and immunity of juvenile broilers. Poult Sci. (2018) 97:3489–93. doi: 10.3382/ps/pey220, PMID: 29897509

[178] Yin FG Liu YL Yin YL Kong XF Huang RL Li TJ . Dietary supplementation with Astragalus polysaccharide enhances ileal digestibilities and serum concentrations of amino acids in early weaned piglets. Amino Acids. (2009) 37:263–70. doi: 10.1007/s00726-008-0142-6, PMID: 18622730

[179] Li S Ren L Zhu X Li J Zhang L Wang X . Immunomodulatory effect of γ-irradiated Astragalus polysaccharides on immunosuppressed broilers. Anim Sci J. (2019) 90:117–27. doi: 10.1111/asj.13133, PMID: 30456927

[180] Qiao Y Liu C Guo Y Zhang W Guo W Oleksandr K . Polysaccharides derived from Astragalus membranaceus and Glycyrrhiza uralensis improve growth performance of broilers by enhancing intestinal health and modulating gut microbiota. Poult Sci. (2022) 101:101905. doi: 10.1016/j.psj.2022.101905, PMID: 35576745 PMC9117935

[181] Hao X Lin H Lin Z Yang K Hu J Ma Z . Effect of Dietary Astragalus polysaccharides (APS) on the Growth Performance, Antioxidant Responses, Immunological Parameters, and Intestinal Microbiota of Coral Trout (Plectropomus leopardus). Microorganisms. (2024) 12:1980. doi: 10.3390/microorganisms12101980, PMID: 39458289 PMC11509791

[182] Yang J Sun Y Wang Q Yu S Li Y Yao B . Astragalus polysaccharides-induced gut microbiota play a predominant role in enhancing of intestinal barrier function of broiler chickens. J Anim Sci Biotechnol. (2024) 15:106. doi: 10.1186/s40104-024-01060-1, PMID: 39103958 PMC11302362

